# Dectin-1 stimulation promotes a distinct inflammatory signature in the setting of HIV-infection and aging

**DOI:** 10.18632/aging.204927

**Published:** 2023-08-21

**Authors:** Archit Kumar, Jiawei Wang, Allen Esterly, Chris Radcliffe, Haowen Zhou, Brent Vander Wyk, Heather G. Allore, Sui Tsang, Lydia Barakat, Subhasis Mohanty, Hongyu Zhao, Albert C. Shaw, Heidi J. Zapata

**Affiliations:** 1 Yale School of Medicine, Section of Infectious Diseases, Department of Internal Medicine, New Haven, CT 06520-8022, USA; 2 Yale University Program on Aging, Yale University, New Haven, CT 06520-8022, USA; 3 Interdepartmental Program in Computational Biology and Bioinformatics, Yale University, New Haven, CT 06520-8022, USA; 4 Yale University, Yale AIDS Care Program, New Haven, CT 06520-8022, USA

**Keywords:** immune response, innate immune cells, HIV-infection, aging, dectin-1

## Abstract

Dectin-1 is an innate immune receptor that recognizes and binds β-1, 3/1, 6 glucans on fungi. We evaluated Dectin-1 function in myeloid cells in a cohort of HIV-positive and HIV-negative young and older adults. Stimulation of monocytes with β-D-glucans induced a pro-inflammatory phenotype in monocytes of HIV-infected individuals that was characterized by increased levels of IL-12, TNF-α, and IL-6, with some age-associated cytokine increases also noted. Dendritic cells showed a striking HIV-associated increase in IFN-α production. These increases in cytokine production paralleled increases in Dectin-1 surface expression in both monocytes and dendritic cells that were noted with both HIV and aging. Differential gene expression analysis showed that HIV-positive older adults had a distinct gene signature compared to other cohorts characterized by a robust TNF-α and coagulation response (increased at baseline), a persistent IFN-α and IFN-γ response, and an activated dendritic cell signature/M1 macrophage signature upon Dectin-1 stimulation. Dectin-1 stimulation induced a strong upregulation of MTORC1 signaling in all cohorts, although increased in the HIV-Older cohort (stimulation and baseline). Overall, our study demonstrates that the HIV Aging population has a distinct immune signature in response to Dectin-1 stimulation. This signature may contribute to the pro-inflammatory environment that is associated with HIV and aging.

## INTRODUCTION

Dectin-1(CLEC7A) is a member of the C-type lectin receptor family that recognizes carbohydrate structures on pathogens, with Dectin-1 specifically recognizing and binding β-1,3/1,6--glucans [1–3], a component of the fungal cell wall. Dectin-1, originally identified as “dendritic-cell-associated C-type lectin-1” [4] is primarily expressed on myeloid cells, including dendritic cells, monocytes, macrophages, and neutrophils. Dectin-1 expression has also been noted on non-immune cells such as lung epithelium [5] and intestinal M cells [6]. Through the recognition of β-glucans, Dectin-1 can recognize human pathogens that are particularly important causes for infection in immunocompromised hosts, such as *Aspergillus*, *Candida*, *Coccidioides*, *Talaromyces*, and *Pneumocystis* [1, 2]. Binding and stimulation of Dectin-1 leads to phosphorylation of its ITAM motif, subsequent downstream signaling through the Syk/CARD9 pathway that ultimately results in NF-κB activation and inflammatory cytokine production. Dectin-1 activation also induces activation of the NLRP3 inflammasome and a Th-17 response [1]—playing a major role in anti-fungal immunity. Additionally, Dectin-1 may also recognize other microbial pathogens such as bacteria (*Salmonella*), mycobacteria [7, 8] as well as endogenous ligands [9] that may contribute to age-related chronic inflammation.

Invasive fungal infections in humans carry unacceptably high rates of mortality that can exceed 50% despite anti-fungal therapies. Patients with HIV-infection [10], and increased age [11, 12] are at high risk but the effects of HIV-infection and age on Dectin-1 function remain unknown. Consequently, we evaluated Dectin-1 function in a cohort of HIV-infected and uninfected young and older adults. Peripheral mononuclear cells (PBMCs) were stimulated with whole glucan particles (1,3/1,6-β-glucan from *S. cerevisiae*) which specifically stimulate Dectin-1 and lack TLR stimulating activity. Using multicolor flow cytometry, we evaluated monocytes, the most abundant innate cells in the peripheral blood functioning as antigen presenting cells that may differentiate into macrophages and dendritic cells at sites of inflammation. Monocytes can be subdivided into the following subsets as defined by their expression of CD14 and CD16: classical (CD14+CD16lo), inflammatory (CD14+CD16+, known to be expanded in the setting of aging and HIV infection) [13], and non-classical (CD14lo CD16hi) subset (also increased with HIV disease). Monocytes can also be further defined by the expression of activation markers such as CD11b of which the expression is also increased in the setting of aging and HIV infection [13–17]. Dectin-1 function was also studied in human dendritic cells which are potent antigen presenting cells found in the peripheral blood and lymphoid tissue. Dendritic cells can be subdivided into myeloid dendritic cells (CD11c+, CD123-; mDCs), and plasmacytoid dendritic cells (CD11c-, CD123+; pDCs) via expression of CD11c and CD123 [18] as previously described. Finally, we performed RNA-seq analysis on sorted inflammatory monocytes to elucidate the effects of age and HIV infection on transcriptomic signatures at baseline and upon Dectin-1 activation.

## RESULTS

We recruited a total of 81 HIV-negative and HIV-positive participants, divided between younger and older adults (as noted in the Methods); the characteristics of enrolled individuals are shown in Table 1. The HIV-positive and HIV-negative groups were comparable in age and gender distribution, incidence of comorbidities such as diabetes, metabolic syndrome, cardiovascular disease and pulmonary disease. HIV-positive adults, compared to the HIV-negative cohort, had higher rates of recreational drug use, number of co-morbidities and differed in distribution of self-reported race and Hispanic ethnicity. Nearly all of the HIV-infected cohort were on antiretroviral therapy (ART) and had CD4 counts >200/mm^3^. However, to account for the effects of differing years with HIV-infection, percent lifespan with HIV-infection (since diagnosis) was calculated for each HIV-infected subject and is represented as a median with an interquartile range (25 percentile, 75 percentile). Of note, there were four congenitally HIV-infected subjects, all in the young group. Please note that these participants are further stratified by age and HIV status in Supplementary Table 3.

**Table 1:** Descriptive statistics stratified by HIV status (N=81).

		**Monocytes**			**Dendritic cells**		
		**HIV positive**	**HIV negative**	**p-value**	**HIV positive**	**HIV negative**	**p-value**
**Total**		36	45		27	39	
**Age Group**	Younger	14 (38.9)	21 (46.7)	0.483	10 (37.0)	21 (53.9)	0.215
	Older	22 (61.1)	24 (53.3)		17 (63.0)	18 (46.2)	
**Gender**	Male	21 (58.3)	20 (44.4)	0.214	16 (59.3)	19 (48.7)	0.399
	Female	15 (41.7)	25 (55.6)		11 (40.7)	20 (51.3)	
**Race/Ethnicity**	Hispanic	10 (27.8)	3 (6.7)	0.014^1^	5 (18.5)	1 (2.6)	0.038^1^
	White	14 (38.9)	33 (73.3)	0.001^1^	13 (48.1)	32 (82.1)	<.001^1^
	Black	15 (41.7)	6 (13.3)		9 (33.3)	3 (7.7)	
	Asian	0 (0)	4 (8.9)		0 (0)	4 (10.3)	
	Other	7 (19.4)	2 (4.4)		5 (18.5)	0 (0)	
**Smoking**		9 (25)	5 (11.1)	0.14^1^	7 (25.9)	3 (7.7)	0.077^1^
**Recreational Drugs**		11 (30.6)	2 (4.4)	0.002^1^	9 (33.3)	1 (2.6)	<.001^1^
**History of Fungal Disease**		4 (11.1)	0 (0)	0.04^1^	7(25.9)	0 (0)	0.001
**Number of Comorbidities**	0	0 (0)	6 (13.3)	<.001^1^	0 (0)	6 (15.4)	0.024^1^
	1-3	10 (27.8)	17 (37.8)		7 (25.9)	17 (43.6)	
	4-7	19 (52.8)	12 (26.7)		11 (40.7)	11 (28.2)	
	>7	7 (19.4)	10 (22.2)		9 (33.3)	5 (12.8)	
History of positive PPD / Quantiferon*		6 (16.7)	2 (4.5)	0.131^1^	4 (14.8)	2 (5.3)	0.224^1^
**Diabetes**		8 (22.2)	11 (24.4)	0.815	6 (22.2)	7 (17.9)	0.458
**Metabolic Syndrome**		5 (13.9)	11 (24.4)	0.273^1^	4 (14.8)	7 (17.9)	1.000
**Cardiovascular Disease**		10 (27.8)	12 (26.7)	0.911	10 (37)	9 (23.1)	0.218
**Pulmonary Disease**		11 (30.6)	12 (26.7)	0.700	7 (25.9)	9 (23.1)	0.791
**CD4 count >200**		35 (97.2)			26 (96.3)		
**On ART**		34 (94.4)			25 (92.6)		
**HIV VL > 100**		4 (11.1)			7 (13.5)	2 (7.4)	
**% life span HIV**		26.1 (18.4-44.6)^2^			34.3 (12.1-49.1)^2^		

Demographic information for HIV-positive and HIV-negative cohorts. Metabolic syndrome = DM + HTN + HLD, PPD = purified protein derivative. All values are n (%). Unless otherwise noted, significance was evaluated using a chi-square test. P values less than or equal to 0.05 were considered significant. The age groups include younger = 21-35, and (≥ 60 years) for most experiments (Monocytes). However, the age groups were extended to 21-40 and (≥ 50 years) for the Dendritic cell experiments.

^1^Significance assessed using Fisher’s Exact Test.

^2^The value of % life span with HIV (since diagnosis) shown in the table represents an interquartile range, with the median (25 percentile, 75 percentile).

**Figure 1:**
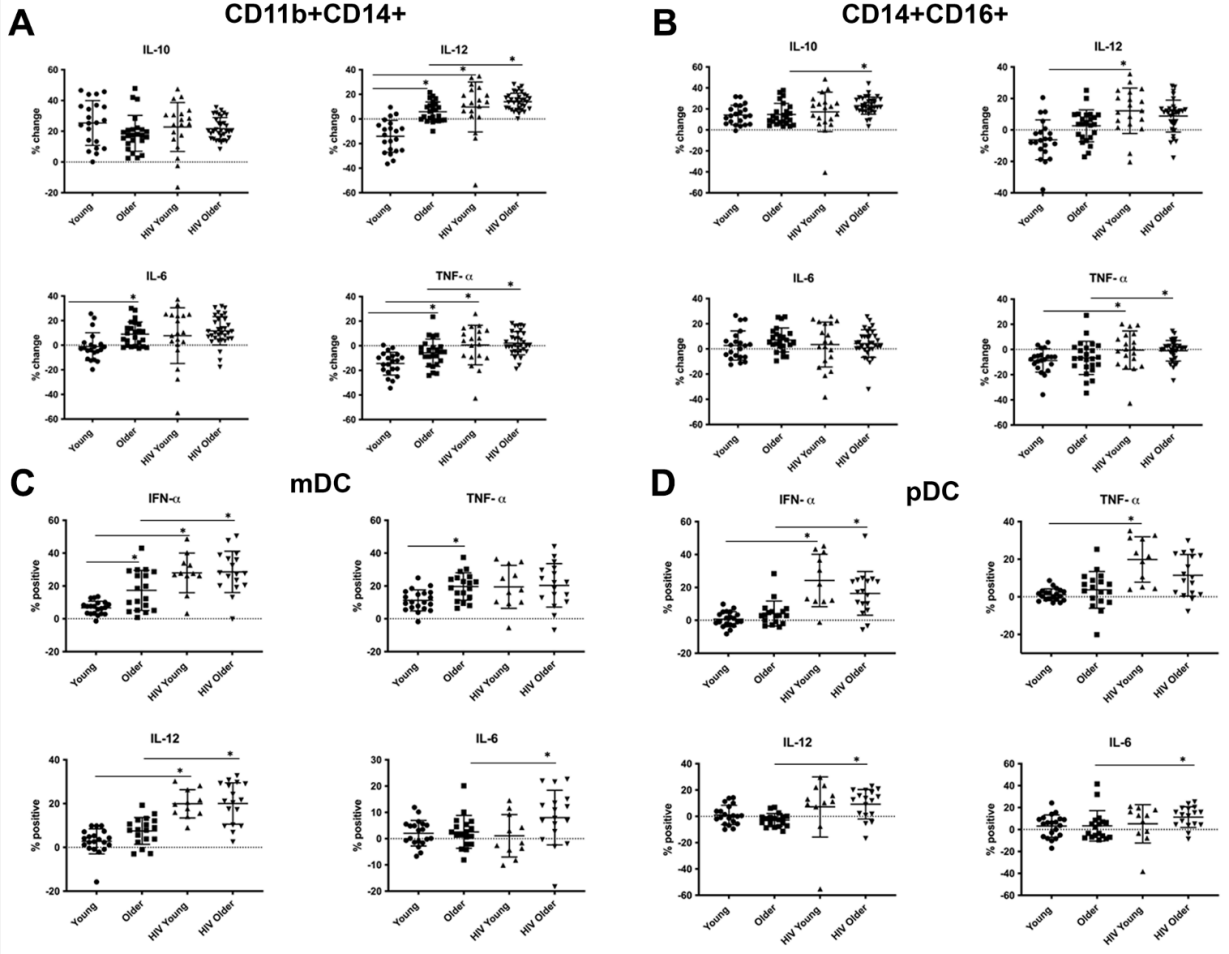
**Effects of age and HIV-infection on Dectin-1 induced cytokine production in monocytes and dendritic cells.** HIV-negative young adults (Young) (n=21), HIV-negative older adults (Older) (n= 24), HIV-positive young adults (HIV-Young) (n =14), and HIV-positive older adults (HIV-Older) (n = 22). Dot plots showing percent change in production of interleukin IL-10, IL-12 (p70 isoform), IL-6, and tumor necrosis factor-α (TNF-α) compared to baseline after stimulation with whole glucan particles (WGP or 1,3/1,6-β-glucan). The following comparisons indicated by asterisks were statistically significant using a Wilcoxon two-sample test with *t* approximation, which were then adjusted with a false discover rate (FDR) calculation for multiple comparisons. (**A**) Total cytokine production in CD11b+ activated monocytes. Young vs. Older: IL-12 (p= 0.00065), IL-6 (p= 0.006), TNF-α (p= 0.026), Young vs. HIV-Young: IL-12 (p=0.011), TNF-α (p=0.016), Older vs. HIV-Older: IL-12 (p=0.01), TNF-α (0.0057). (**B**) Total cytokine production in Inflammatory (CD14+CD16+) monocytes. Young vs. HIV-Young: IL-12 (p=0.0199), TNF-α (p=0.024), Older vs. HIV-Older: IL-10 (p= 0.011), TNF-α (0.032). (**C**) Total cytokine production in myeloid dendritic cells. HIV-negative young adults (n=21), HIV-negative older adults (n= 18), HIV-positive young adults (n =10), and HIV-positive older adults (n = 17). Dot plots showing percent change in production of interleukin IFN-α, TNF-α, IL-12, and IL-6 compared to baseline after stimulation with whole glucan particles (WGP or 1,3/1,6-β-glucan) (labeled as percent positive). The following comparisons indicated by asterisks were statistically significant using a Wilcoxon two-sample test with *t* approximation, which were then adjusted with a false discovery rate (FDR) calculation for multiple comparisons. i) IFN-α, Young vs. Older (p=0.0373), Young vs. HIV-Young (p=0.0004), Older vs. HIV-Older (p=0.011). ii) TNF-α, Young vs. Older (p=0.023), iii) IL-12, Young vs. HIV-Young (p=0.0001), Older vs. HIV-Older (p=0.001) iiii) IL-6, Older vs. HIV-Older (p=0.035). (**D**) Total cytokine production in plasmacytoid dendritic cells. HIV-negative young adults (n=21), HIV-negative older adults (n= 18), HIV-positive young adults (n =10), and HIV-positive older adults (n = 17). Dot plots showing percent change in production of interleukin IFN-α, TNF-α, IL-12, and IL-6 compared to baseline after stimulation with whole glucan particles (WGP or 1,3/1,6-β-glucan) (labeled as percent positive). The following comparisons indicated by asterisks were statistically significant using a Wilcoxon two-sample test with *t* approximation, which were then adjusted with a false discovery rate (FDR) calculation for multiple comparisons. i) IFN-α, Young vs. HIV-Young (p=0.0003), Older vs. HIV-Older (p=0.011). ii) TNF-α, Older vs. HIV-Young (p=0.0085), iii) IL-12, Older vs. HIV-Older (p=0.0011). iiii) IL-6, Older vs. HIV-Older (p=0.035). All other comparisons were not significant.

Freshly isolated PBMCs were stimulated with whole glucan particles (1,3/1,6-β-glucan, WGP Dispersible), a specific Dectin-1 agonist [19] and analyzed via intracellular cytokine staining (ICS) and multicolor flow cytometry. A soluble form of WGP that binds to the Dectin-1 receptor without activation was used as a negative control as noted in the Methods. The soluble form of WGP did not elicit any cytokine production (Supplementary Figure 14). We determined intracellular IL-10, IL-12, IL-6 and TNF-α production in classical (CD14+CD16lo), inflammatory (CD14+CD16+), activated (CD11b+CD14+) and non-classical monocytes (CD14loCD16H) monocytes [15]. In activated CD11b+ CD14+ monocytes, we found an age-associated increase in Dectin-1-induced IL-6, IL-12 and TNF-α production in older, compared to young, HIV-negative adults. Furthermore, TNF-α and IL-12 production in these activated monocytes was increased in both young and older adults with HIV infection, when compared to HIV-negative adults, respectively (Figure 1A). Robust IL-10 production appeared comparable in all cohorts. These results in CD11b+ monocytes reflect an age- and HIV-associated heightened activation of Dectin-1 function. CD14+ CD16+ inflammatory monocytes also showed an HIV-associated increase in TNF-α production (HIV-positive vs. HIV negative individuals) in both young and older adults. An HIV-associated increase in IL-12 levels was also noted in HIV-positive young, when compared to HIV-negative young individuals (Figure 1B). Classical monocytes (CD14+CD16lo) demonstrated an age-associated increase in production of IL-12 and IL-6, but in contrast to CD14+ CD11b+ or inflammatory monocytes a statistically significant effect of HIV infection was not found (Supplementary Figure 2A). In contrast, non-classical CD14+CD16H monocytes from HIV-negative adults showed a significant age-associated decrease in intracellular IL-12 levels, with a trend of decreased TNF-α production that did not reach significance. Cytokine production of IL-12 and TNF-α in HIV-positive young and older adults was comparable to the young HIV-negative cohort (Supplementary Figure 2A, 2B). Taken together, these results indicate that activated CD11b+ and inflammatory monocytes show a HIV-associated increase in TNF-α and IL-12 production. In the CD11b+ activated subset, we also observed an age associated increase in IL-12, TNF-α and IL-6 production. Baseline cytokine data shown in Supplementary Figure 15, show increased basal levels of TNF-α, IL-12, IL-6, and IL-10 in the CD14+CD16+ in the setting of HIV-infection. This trend is also noted in the CD11b+CD14+ with the exception of TNF-α which showed decreased levels in the setting of HIV-infection. General trends of cell percentages for monocytes before and after stimulation are shown in Supplementary Figure 13.

**Figure 2:**
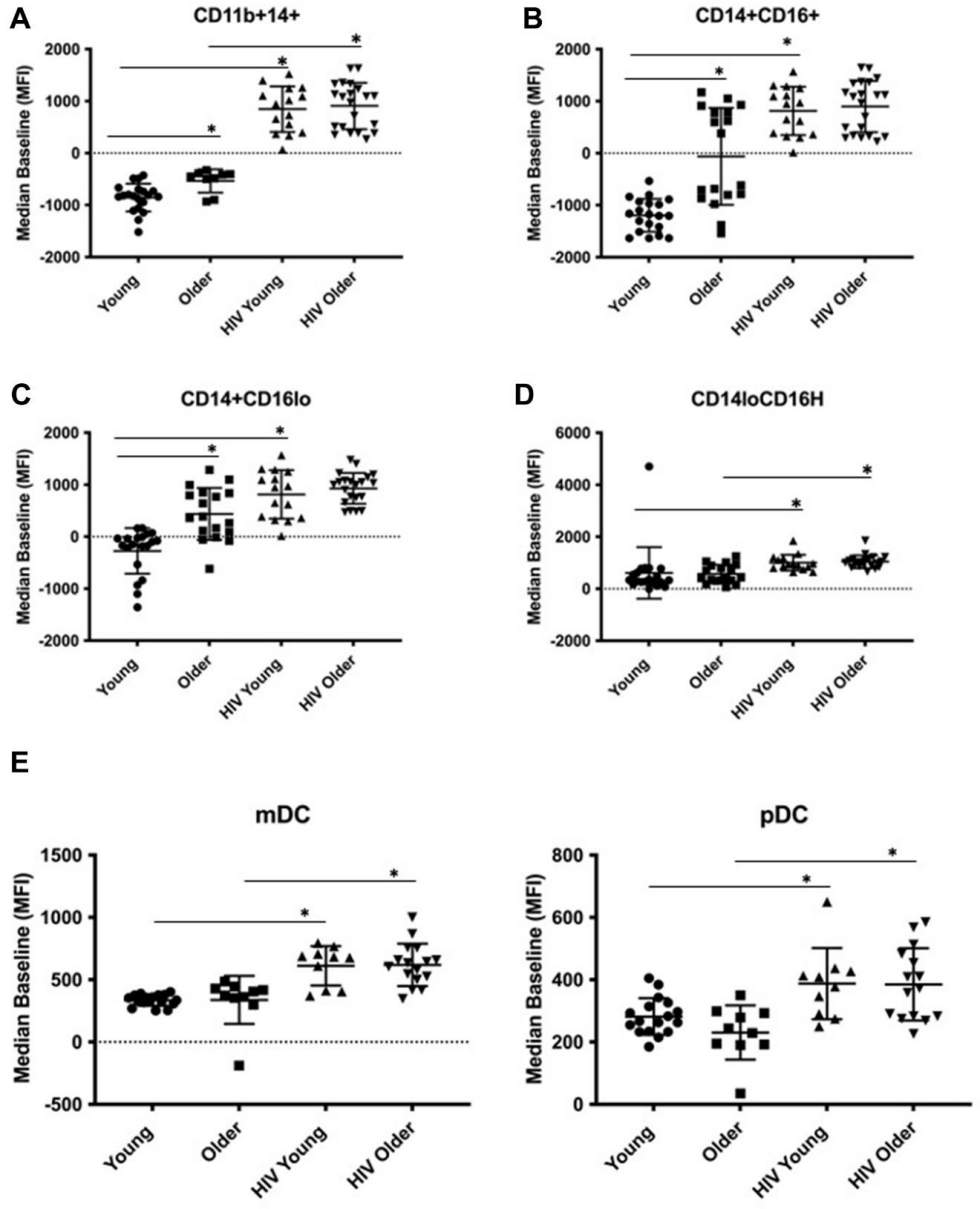
**Dectin-1 surface expression in monocytes and dendritic cells.** The cohort consists of the following: Monocytes: [HIV-negative young adults (Young) (n=21), HIV-negative older adults (Older) (n= 24), HIV-positive young adults (HIV-young) (n =14), and HIV-positive older adults (HIV-Older) (n = 22)]. Dendritic cells: [Young (n=21), Older (n=18), HIV-Young (n=10), HIV-Older (n= 17)]. Dot plots showing the Median of the Mean Fluorescence Intensity (MFI) of Dectin-1 surface expression. The following comparisons indicated by asterisks were statistically significant using a Wilcoxon two-sample test with *t* approximation, which were then adjusted with a false discover rate (FDR) calculation for multiple comparisons. (**A**) Activated Monocytes (CD11b+ CD14+). Young vs. Older (p= 0.026), Young vs. HIV-Young (p=0.001), Older vs. HIV-Older, (p=0.005). (**B**) Inflammatory Monocytes (CD14+CD16+). Young vs. Older (p= 0.003), Young vs. HIV-Young (p=0.001). (**C**) Classical Monocytes (CD14+CD16lo). Young vs. Older (p= 0.0039), Young vs. HIV-Young (p=0.0026). (**D**) Non-classical monocytes (CD14lo, CD16H). Young vs. HIV-Young (p=0.011), Older vs. HIV-Older, (p=0.048). (**E**) Dectin-1 Surface Expression in Myeloid Dendritic cells (mDC) and Plasmacytoid Dendritic Cells (pDC). i). mDC, Young vs. HIV-Young (p=0.0002), Older vs. HIV-Older (p=0.0018). ii). pDC, Young vs. HIV-Young (p=0.012), Older vs. HIV-Older (p=0.0085).

To study the basis for altered Dectin-1-dependent cytokine production, we evaluated the effects of age and HIV infection on Dectin-1 surface expression in monocyte subsets (Figure 2). We found significantly increased surface expression of Dectin-1 in both older adults and HIV-infected individuals in all monocyte populations with the exception of the nonclassical (CD14+ CD16H) where we only saw an HIV-associated difference in Dectin-1 surface expression. For HIV-positive young adults, Dectin-1 expression was increased in all monocyte subsets compared to HIV-negative young individuals, and we found a similar increase in HIV-positive older, compared to HIV-negative older adults that was statistically significant for the CD14+ CD11b+ and CD14+ CD16H subsets. For the activated CD14+ CD11b+ monocytes, in which we saw both an age and HIV-associated increase in Dectin-1 surface expression, we built a multivariable regression model to incorporate the effects of clinical covariates (Table 2). In the unadjusted model, both age and HIV-infection were significant and non-interacting contributors. However, the addition to the model the co-variates of recreational drug use, history of fungal disease, number of medical co-morbidities, and percent lifespan with HIV led to a loss of significance for age. In the fully adjusted model, Dectin-1 surface expression was significantly associated with HIV-infection, percent lifespan with HIV, recreational drug use, and number of co-morbidities. When IL-12 in CD14+CD11b+ activated monocytes was evaluated in a multivariable regression model (Supplementary Table 4) both age and HIV-infection remained significant contributors even when adjusted for drug use, fungal disease, co-morbid conditions, and % lifespan with HIV. Only age remained significant when TNF-α was evaluated in activated monocytes within an adjusted model.

**Table 2:** Dectin-1 surface expression in activated monocytes is significantly associated with HIV-infection, recreational drug use, co-morbid conditions, and % lifespan with HIV.

	**Unadjusted model**			Adjusted model*		
	**Parameter estimate**	**Standard error**	**P-value**	**Parameter estimate**	**Standard error**	**P-value**
Age (60+ vs 21-35)	321.5	128.9	0.013	145.3	138.5	0.294
HIV (Positive vs Negative)	1741.1	124.4	0.000	1271.0	149.7	0.000
Age x HIV interaction	-322.9	193.1	0.094	-100.5	172.5	0.560
Recreational Drug Use				338.3	124.1	0.006
Fungal Disease				-204.5	222.6	0.358
Co-morbid conditions				147.3	66.3	0.026
% lifespan with HIV				9.5	3.0	0.001
**Least squares means with 95% confidence intervals for interaction results**						
**Age/HIV status**	**Unadjusted means**			**Adjusted means**		
21-35 and HIV negative	-855.7 (-996.5,-714.9)			-640.9 (-798.7,-483)		
21-35 HIV positive	885.4 (686.3,1084.5)			630.1 (423.4,836.8)		
60+ HIV negative	-534.2 (-744.1,-324.3)			-495.6 (-704.5,-286.6)		
60+ HIV positive	884 (684.9,1083.1)			674.9 (478.9,870.8)		

Table outlining the multiple variable linear regression model for Dectin-1 surface expression, with the following variables (Age, HIV status, Age/HIV interaction term), and the following covariates (history of recreational drug use, history of fungal infection, number of co-morbid conditions, and % HIV life span).

*In addition to age, HIV Status, and their interaction, the adjusted model has covariates for the number of comorbidities, percentage (%) of life with HIV, history of fungal disease, and recreational drug use.

We also examined the response of Dectin-1 stimulation in dendritic cells (DCs) in the context of aging and HIV-infection. Lineage negative and HLA-DR positive cells were separated into CD11c+, CD123- (mDCs), and CD11c-, CD123+ (pDCs) [18]. We determined intracellular production of IFN-α, TNF-α, IL-6 and IL-12 in both subsets following WGP stimulation. mDCs (Figure 1C) showed a significant age-associated increase in IFN-α and TNF-α production with Dectin-1 stimulation, and an HIV-associated increase in IFN-α and IL-12 production in both young and older adults. IL-6 production was comparable among these groups except for a significant increase in HIV-positive versus HIV-negative older adults. Cytokine production in pDCs (Figure 1D) was comparable in HIV-negative young and older adults. However, there was a significant HIV-associated increase in IFN-α, TNF-α (in HIV-positive young) and IL-6 and IL-12 (in HIV-positive older) production. Dectin-1 surface expression levels in mDCs and pDCs showed a significant HIV-associated increase when compared to HIV-negative adults (Figure 2E). Baseline cytokine data shown in Supplementary Figure 16 show an increase in basal levels of TNF-α and IL-12 in mDCs in the HIV-infected cohorts. IL-12 basal levels were also noted to be increased with HIV-infection in the pDC population. Basal IL-6 levels interestingly were increased in the young HIV-negative cohort compared to other groups. Basal IFN-α were comparable between all groups in both mDCs and pDCs. Overall, our findings show that Dectin-1-dependent type I interferon production is increased with HIV infection in mDCs and pDCs from both young and older adults, with age-related increases in IFN-α and TNF-α found in mDCS from HIV-negative older adults.

### Effects of age and HIV infection on transcriptomic profiling identifies differentially expressed genes in inflammatory monocytes from the different cohorts

We investigated the basis for the age and HIV-associated alterations in Dectin-1 function, focusing on the CD14+ CD16+ inflammatory monocyte subset, which overlaps with the CD11b+ subset and is expanded in the setting of aging and HIV-infection [13]. Sorted CD14+ CD16+ monocytes from 8 HIV-positive and 11 HIV-negative young and older adults were stimulated with WGP and subjected to RNA-seq analysis. The clinical characteristics for all the enrolled individuals used for the RNAseq study can be reviewed in Supplementary Table 2. Of note, Young* and Older* refer to cohorts with increased counts of co-morbidities (please refer to Methods).

**Figure 3:**
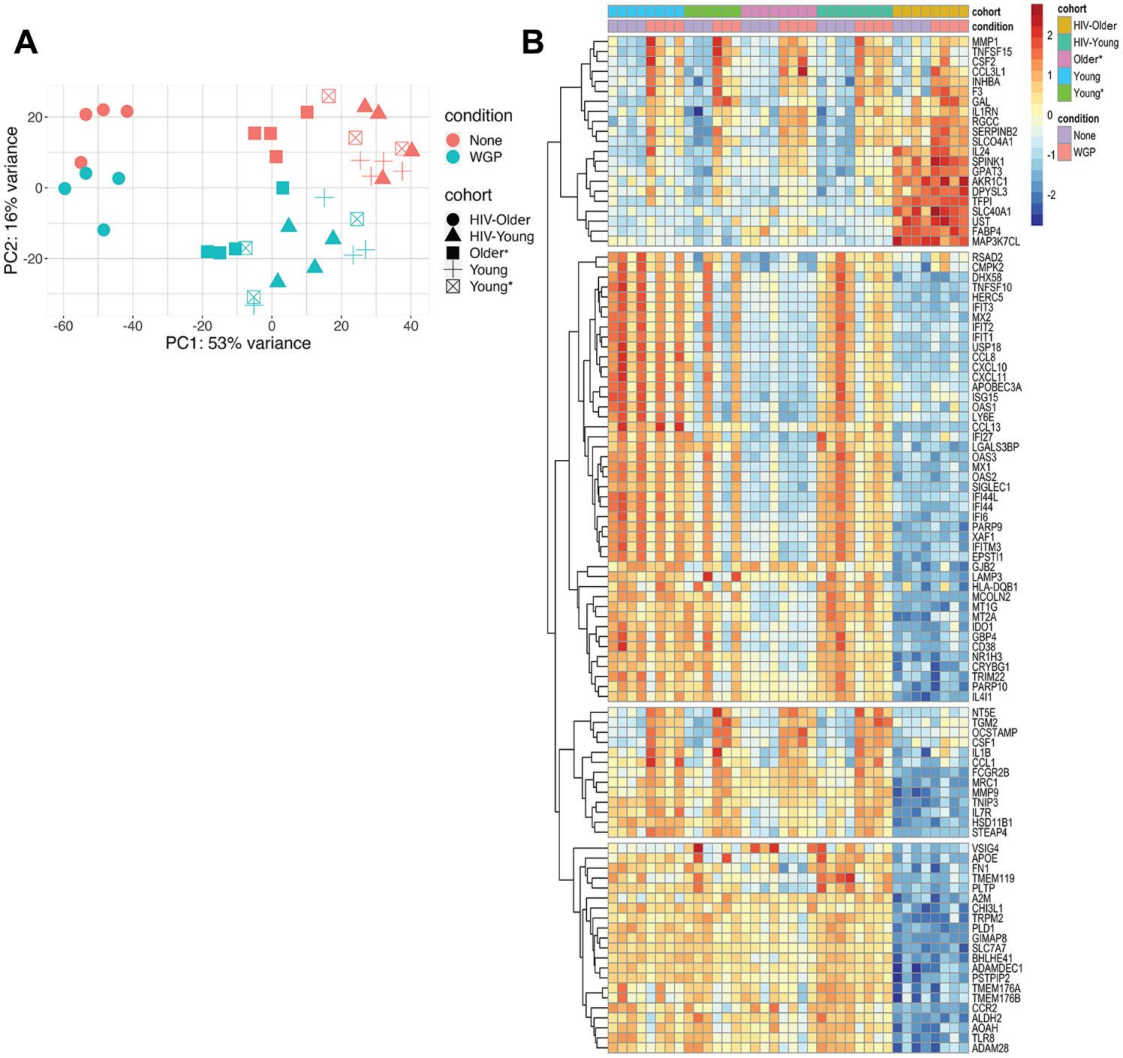
(**A**) Principal component analysis highlights transcriptional differences between the HIV-positive older adults and all other cohorts. Principal component analysis was performed to identify age, HIV, and Dectin-1 stimulation associated differences among Inflammatory (CD14+CD16+) monocytes. Dectin-1 stimulated vs. unstimulated Inflammatory monocytes were separated with 16% variance on PC2, and monocytes from HIV older adults vs all other cohorts are separated by 53%. [HIV-Older n= 4, HIV-Young n =4, Young = 3, Young* = 4, Older = 3]. Please note that the asterisk (*) indicates the presence of co-morbidities. None = Unstimulated, WGP= Dectin-1 stimulation. (**B**) Heatmap of top 100 variable genes. A total of 314 (Young (Y)), 541 (Young * (Y*)), 402 (Older (O)), 860 (HIV-Young (HIV-Y)), and 156 (HIV Older (HIV-O)) unique DEG (differentially expressed genes) were noted for each of the cohorts respectively. The genes with a fold change of (FC) 1.2 and FDR < 0.1 were defined as differentially expressed genes (DEGs). This heat map notes differences in HIV Older vs. all other cohorts–the effect of stimulation is seen in the second row (Pink column). The heatmap was constructed using Pheatmap. The transcripts were normalized using variance stabilizing transformation function. The color represents relative expression of transcripts that covary across cohorts and condition.

Principal component analysis explained 69% of the variance; baseline and Dectin-1-stimulated inflammatory monocytes were clearly distinguished on PC2, while monocytes from HIV-positive and HIV-negative older adults were separated on PC1 (accounting for 53% of the variance). Notably, while samples from young HIV-positive vs. young HIV-negative adults did not show obvious separation (including HIV-negative young adults with increased comorbid medical conditions), there was a clear distinction between HIV-positive and HIV-negative older adults both at baseline and following Dectin-1 stimulation. Notably, HIV-older adults showed distinct separation from the other groups (Figure 3A).

Upon WGP stimulation, we identified 314, 541, 402, 860, and 156 differentially expressed genes (DEGs) relative to baseline among Young, Young* (with increased medical comorbidities), Older, HIV-Young, and HIV-Older, respectively. A heat map of the top 100 variable genes among the cohorts is shown in Figure 3B, and shows baseline and post-stimulation differences in the HIV-negative young cohorts with and without increased medical comorbidities. Substantial alterations in gene expression were found for both HIV-negative and HIV-positive older, compared to young individuals.

### Dectin-1 stimulation elicits both distinct and common transcriptional gene signatures in each cohort

**Figure 4:**
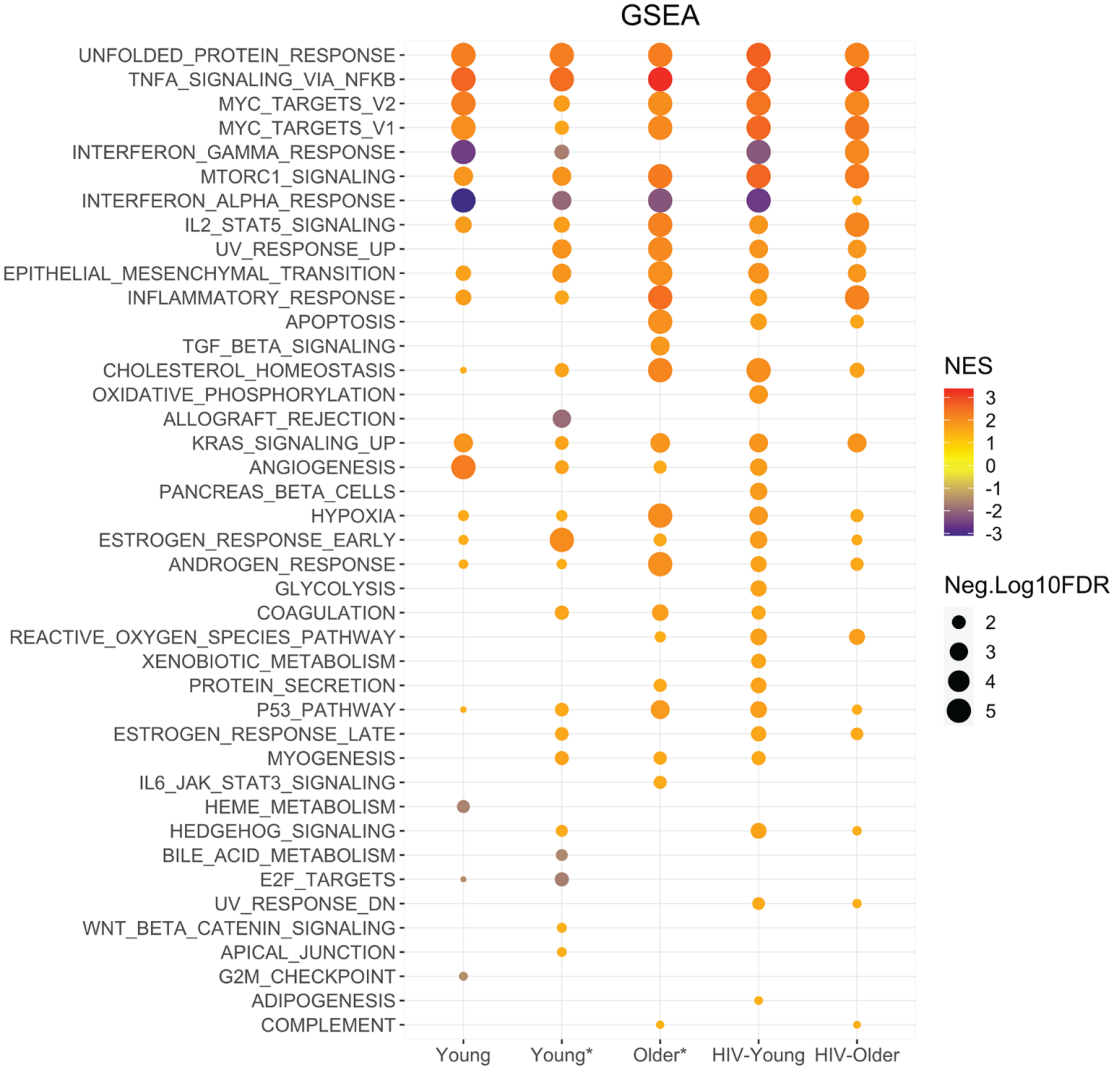
**Enrichment analysis highlights unique signatures with Dectin-1 stimulation across cohorts.** Functional enrichment was performed using the Broad institutes Hallmark gene set enrichment analysis. GSEA performed using Hallmark gene sets for each cohort. The dot graph represents the significant Hallmark pathways identified in WGP stimulated Inflammatory monocytes of the respective cohort. The pathways with FDR of <5% were considered significant. For graphical representation, FDR values with 0 were adjusted to 0.00001. The size of the node represents -Log10(FDR) and color of node represent normalized enrichment score. NES = Normalized enrichment scores.

Functional enrichment was performed on all the cohorts that were Dectin-1 stimulated. The Hallmark gene set enrichment analysis showed significant enrichment for the inflammatory response, and TNF-α and IL-2/STAT5 signaling in all cohorts in response to Dectin-1 stimulation with increased enrichment noted in the HIV older group and the Older HIV-negative group compared to the young adults (Figure 4). The functional analysis of DEGs using KEGG pathways (Supplementary Figure 3) confirmed significant enrichment of TNF signaling in all cohorts, in agreement with Hallmark gene sets. KEGG analysis also showed upregulation of IL-17 signaling, cytokine-cytokine receptor interactions, NF-κB signaling, complement and coagulation cascades, and MAPK signaling pathways in response to Dectin-1 stimulation in most cohorts (Supplementary Figure 3).

Several metabolic pathways were also strongly induced with Dectin-1 stimulation, including mTORC1 signaling and the hypoxia pathway in all cohorts (Figure 4) with increased enrichment scores noted in the setting of aging and HIV-infection. With respect to the PI3K-Akt signaling pathway; we found diminished in PI3K-Akt signaling in the HIV Older group, that could in part reflect baseline pathway enrichment relative to young HIV-negative adults (Supplementary Figures 3, 6). GSEA baseline analysis also showed upregulation of mTORC1 signaling in the HIV older group that was not seen in any other cohort (indeed downregulation was found in the HIV young group) (Supplementary Figure 7). Finally, the unfolded protein response, indicative of endoplasmic reticulum stress was strongly upregulated in all cohorts with Dectin-1 stimulation (Figure 4). Overall, these findings show strong metabolic signatures that are induced by Dectin-1 stimulation with some baseline activation of MTORC1 noted in the HIV-older cohort.

The HIV-older group in particular showed additional differences in gene expression. In contrast to young adults, both HIV-older and HIV-negative Older* adults failed to upregulate PPAR and Rap1 signaling pathways in response to Dectin-1 stimulation (Supplementary Figure 3). In this regard, KEGG baseline pathway analysis showed that PPAR signaling was already upregulated in the HIV-Older adults (Supplementary Figure 8). Besides regulating cytokines, Dectin-1 stimulation is also known to initiate the production of reactive oxygen species (ROS) [20]. Dectin-1 stimulation of inflammatory monocytes resulted in the significant enrichment of the Hallmark Reactive Oxygen Species pathway among Older, HIV-Older and HIV-young but not young HIV-negative adults (Figure 4), with baseline upregulation of the ROS pathway noted only in HIV-Older adults (Supplementary Figure 7). While cytokine signaling pathways were generally upregulated in stimulated inflammatory monocytes (Supplementary Figure 4A–4D), HIV-Older individuals demonstrated higher baseline levels for the TNF signaling pathway, complement coagulation cascades (Supplementary Figure 4B, 4C) and IL-19 and IL-24 (Supplementary Figure 4A–4D) gene expression compared to other cohorts. IL-7R was upregulated in all cohorts except for the HIV-Older cohort (where upregulation was minimal or absent). Similarly, IL1-β and IL-1A baseline expression was higher in all young cohorts compared to the HIV-positive and HIV-negative older adults, where baseline levels were absent (Supplementary Figure 4A–4D). Induction of IL1-β and IL-1A gene expression with stimulation was seen in all cohorts, although less so in the HIV-Older cohort.

### Dectin-1 induces interferon response in HIV-older adults

GSEA analysis exhibited downregulation of the Interferon-α and -γ response in inflammatory monocytes from all cohorts except for the HIV-positive older cohort (Figure 4), in which upregulation was observed. Interestingly, this gene expression data parallels the increased production of IFN-α that was noted in the HIV Older cohort in both myeloid dendritic cells and plasmacytoid dendritic cells (Figure 1C, 1D). Further examination of these gene sets demonstrated increased basal levels of the IFN-α and -γ response in young adults (both HIV-negative and HIV-positive) that was downregulated (Supplementary Figure 5A) upon Dectin-1 stimulation; in contrast, both HIV-negative and HIV-positive older adults showed decreased basal IFN expression that increased with stimulation. In HIV-older adults (Supplementary Figure 5C) IFN-α and IFN-γ were predicted as an activated upstream regulators of the following IFN inducible genes including; CMPK2, RSAD2, RIPK2, GZMB, CD274 (PDL-1) further confirming downstream signaling. Our RNA-seq analysis was done post hoc of the initial flow cytometry analysis. Therefore, we recruited a subset of subjects to further evaluate the IFN-α and IFN-γ response in monocytes. PBMCs were stimulated with whole glucan particles as noted previously. At baseline we found that both IFN-α and IFN-γ cytokine levels were increased in the HIV-older group compared to young and older HIV-negative subjects (Supplementary Figure 11). Older HIV-negative adults showed a trend of increased basal levels when compared to young HIV-negative adults. With Dectin-1 stimulation we found varying levels of IFN-α production with a trend towards increased expression in the HIV-Older group in the classical monocyte subset. In the other monocyte subsets we found comparable production of IFN-α between the cohorts as there were no significant differences. Interestingly, with Dectin-1 stimulation there was a significant increase noted in IFN-γ production when young HIV-negative adults were compared to HIV-Older adults.

### Activation of dectin-1/CLEC-7 signaling in inflammatory monocytes across cohorts

As expected, Gene set enrichment analysis (GSEA) for REACTOME demonstrated significant enrichment of the reactome Dectin-1 mediated non-canonical NF-κB gene set and Dectin-1 (CLECA7A) signaling pathways gene set in Dectin-1 stimulated Inflammatory monocytes of HIV-Young, HIV-Older, and Older HIV-negative adults (Supplementary Figure 9A). Enrichment scores and q-values were highly significant for the HIV-older and the older (with co-morbidities) (NES>2, q<0.001) HIV-negative group compared to that of HIV-Young adults (Supplementary Figure 9A). Enrichment of Dectin-1 signaling was not significant in the young HIV-negative cohorts (Young and Young*). Although, upregulation of Dectin-1 signaling genes, BCL10 and MALT1 was observed in all cohorts following WGP stimulation of monocytes (Supplementary Figure 9B). Bcl10 and MALT1 both form part of the CARD9 complex that is important for downstream Dectin-1 signaling [1].

### TREM-1 enrichment is associated with Dectin-1 activation

**Figure 5:**
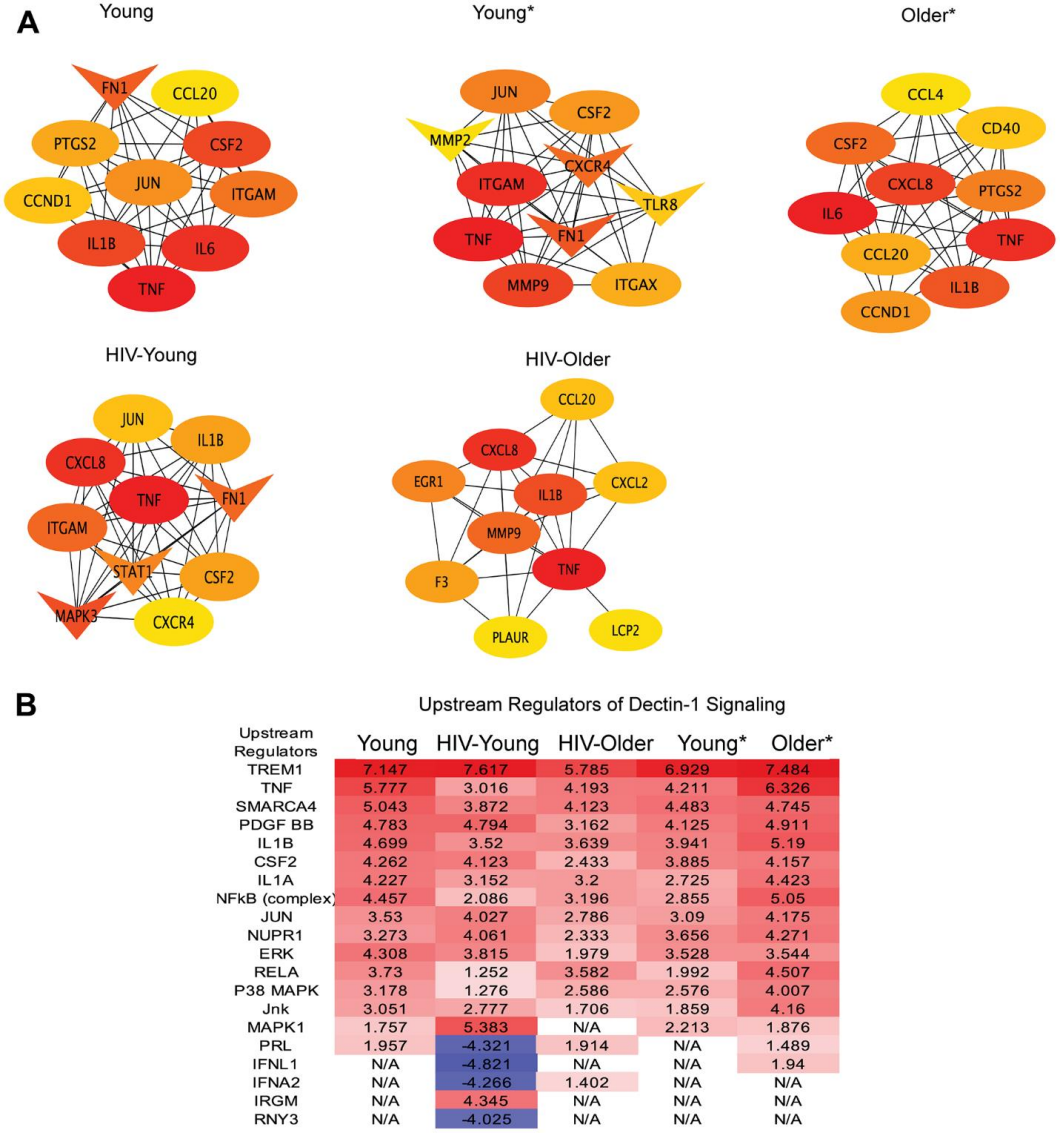
**Upstream regulators and hub genes in Dectin-1 signaling.** (**A**) Hub Gene Networks. Protein-protein interaction network of hub genes identified based on the degree by using CytoHubba plugin of the Cytoscape. Hub genes were identified in all cohorts, Young, Young*, Older*, HIV-Young and HIV-Older. The red color of the node represents a higher degree of interaction, orange represents an intermediate degree of interaction, and nodes with yellow color represent a lower degree of interaction compared to others. Oval nodes reflect upregulated DEGs, triangle shaped nodes represent down-regulated DEGs. (**B**) Upstream regulators of Dectin-1 signaling. DEGs were analyzed for upstream regulators using IPA and top upstream regulators (including transcription factors) with Z-scores cutoff of 4 are listed for all cohorts.

We also carried out GSEA using ImmuneSigDB gene sets, and observed significant enrichment (NES>3, q<0.0001) in Dectin-1 stimulated monocyte subsets (Supplementary Figure 9A) in all groups for Triggering receptor in myeloid cells (TREM-1), an innate immune receptor with unknown activating ligand that has been shown to modulate the inflammatory response [21–24]. Our data also demonstrate that TREM-1 is a prominent upstream regulator of the response to β-D-glucans (Figure 5B) as noted in the next section.

### Hub genes and upstream regulators

The DEGs were further analyzed for protein-protein interaction (PPI) networks by using STRING, and Cytoscape. [The Hub genes were identified from PPI network based on their degree using Cytohubba plugin of Cytoscape.] The top ten hub genes identified in inflammatory monocytes in response to Dectin-1 stimulation are shown in Figure 5A. The common hub genes in many of the cohorts included TNF-α, IL1-β, CSF2 (colony stimulating factor 2), CXCL8 (IL-8), CCL20 (MIP3-α), JUN and ITGAM (CD11b). Hub genes that were unique to the HIV-Older cohort included the coagulation factors F3 (tissue factor) and PLAUR (plasminogen activator), EGR1 (Early growth response protein 1), and LCP2 (Lymphocyte Cytosolic protein 2). Interestingly, the downregulation of STAT1 (Figure 5A) was only observed among HIV-Young individual cohort. These differences in Hub genes imply differential unique protein interactions in each of the cohorts with respect to Dectin-1 signaling.

We also analyzed DEGs for the prediction of upstream regulators by using ingenuity pathway analysis (IPA). The top upstream regulators with Z-scores cutoff of 4 are listed in Figure 5B, while the complete list of the significantly predicted upstream regulators are provided in Supplementary Table 1. The top most activated upstream factors in response to Dectin-1 stimulation include TREM1, TNF, SMARCA4 (actin dependent regulator of chromatin, subfamily A, member 4), IL-1β, NFκB, JUN, ERK, RELA and p38MAPK. The downregulation of the hub gene STAT1 (Figure 5A) along with the inhibition of upstream regulators, IFNL1 and IFNA2 (Figure 5B), suggests the Dectin-1 stimulation inhibits the interferon response in HIV-Young individuals. Both analyses (hub gene analysis and upstream regulators) suggest that TNF-α and IL1-β are central players of Dectin-1 signaling. Upstream regulator analysis shows MAPK signaling in general (p38, JUN, JNK, ERK, MAPK1) plays an important role in Dectin-1 stimulation across cohorts.

### Dectin-1 stimulation of monocytes induces distinct differentiation signatures

We observed significant enrichment of DEGs for transcriptional dysregulation in cancer and hematopoietic cell lineage KEGG gene sets along with significant enrichment for Hallmark epithelial mesenchymal transition (GSEA) in response to Dectin-1 stimulation (Figure 4 and Supplementary Figure 3). The activation of genes such as NR4A3, MET, ZEB1, CSF1, CSF2, CSF3, which can be involved in transcriptional dysregulation in cancer or hematopoietic cell lineage signaling pathways, can also be involved in cell differentiation [25–27]. Therefore, we further investigated GO terms that could be associated with monocyte differentiation.

**Figure 6:**
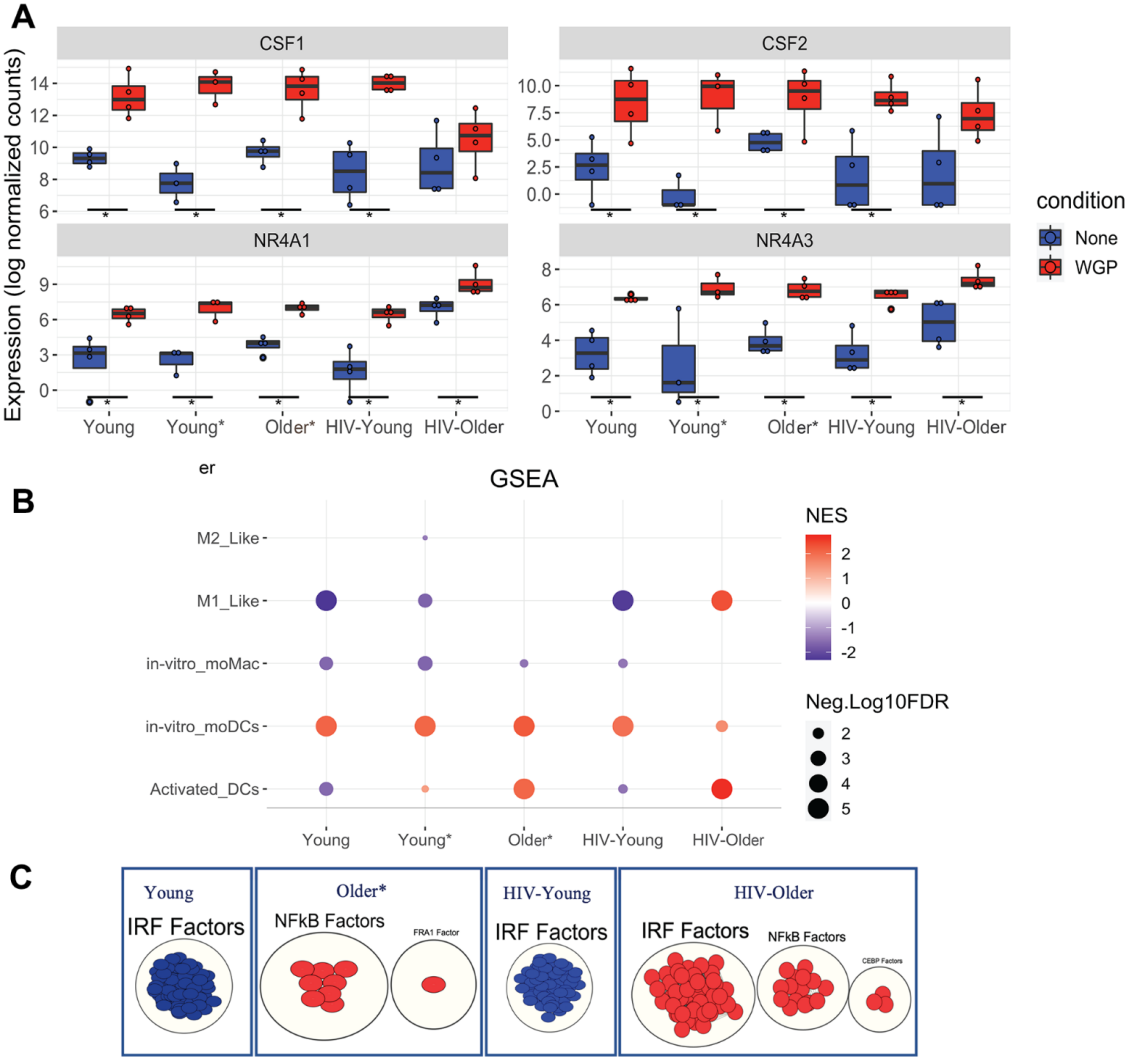
**Dectin-1 stimulation leads to distinct signatures of differentiation.** GSEA was performed on normalized counts obtained from Dectin-1 stimulated Inflammatory monocytes using previously defined gene sets for *in vitro* moDCs and Activated DCs. (**A**) Box plots of CSF1, CSF2, NR4A1, and NR4A3. The symbol * represents significant differentially expressed genes with fold cutoff of 1.2, and q value < 0.1. (**B**) Gene enrichment analysis for monocyte differentiation was performed. The plot represents normalized enrichment scores and FDR (q-values) obtained for each cohort. For graphical representation, FDR values with 0 were adjusted to 0.00001 and FDR of <5% was considered significant for the enrichment of a particular term. (**C**) Transcription factor analysis of the significantly enriched activated DCs gene sets using gProfiler and visualization by Cytoscape. * Significantly differentially expressed genes with FC cut off 1.2 and padj <0.1.

During infection, monocytes can terminally differentiate either to monocyte derived macrophages (moMac) and/or to monocyte-derived dendritic cells (moDCs). Recently, Boulet et al. 2019 demonstrated the importance of the orphan nuclear receptor NR4A3 in the differentiation of murine moDCs in response to LPS stimulation [25]. In our study, Dectin-1 stimulation of Inflammatory (CD14+CD16+) monocytes resulted in the significant upregulation of the NR4A3 and NR4A1 genes in all cohorts (Figure 6A). The expression of granulocyte-macrophage colony-stimulating factor (GM-CSF or CSF2) and macrophage-CSF (M-CSF or CSF1), required for macrophage/dendritic cell differentiation, were also significantly upregulated in all cohorts except for HIV-Old, where a trend was noted.

Therefore, we explored if Dectin-1 stimulation regulates the differentiation or activation of either macrophages or dendritic cells. Gene set enrichment analysis using previously defined gene sets for monocyte differentiation [28–31], revealed significant enrichment for the *in vitro* monocyte derived dendritic cell gene set in Dectin-1 stimulated inflammatory monocytes of all cohorts (Figure 6B). The *in vitro* monocyte derived macrophage gene set was downregulated in most cohorts. However, Dectin-1 stimulated inflammatory monocytes from HIV-older adults showed significant positive enrichment for activated DC and M1-like gene sets. The activated DC gene set was also found to be significantly enriched in monocytes of young and older adults with co-morbid conditions (and downregulated in young HIV-negative and young HIV-positive adults). The gene sets for activated DCs were further analyzed with transcription factor (TF) analysis using gProfiler (Figure 6C), which demonstrated that the genes for activated DCs were positively regulated by Interferon Regulatory Factors (IRFs) in the HIV Older group, and negatively regulated by IRFs in both the HIV-negative and HIV-positive young adults. NF-κB transcription factors also showed positive regulation in the HIV Older group and in the Older HIV negative group.

Dectin-1 induced transcriptomic changes in inflammatory monocytes suggest the differentiation of CD14+CD16+ monocytes mainly to monocyte derived dendritic cells, as these changes were noted in all the cohorts. However, the transcriptomic profile of the stimulated inflammatory monocytes of HIV-older adults also revealed a distinct gene signature that demonstrated an M1-like phenotype in macrophages, as well as an activated DC signature that was also noted in young and Older HIV-negative adults with co-morbidities. Young HIV-negative adults seemed to skew towards a monocyte-derived dendritic cell signature. Overall, these results demonstrate that unique inflammatory signatures are associated with age, and HIV-infection status. Of note, Venn analysis was also performed to identify which genes were commonly expressed among all cohorts, exclusively expressed, and shared between distinct cohorts (Supplementary Figure 12 and Supplementary Table 5).

## DISCUSSION

To our knowledge, our study is the first to evaluate the specific function of Dectin-1 in the setting of aging and HIV-infection. A few themes emerged from our studies. Stimulation of both monocytes and dendritic cells with β-D-glucans led to a more pro-inflammatory phenotype in both monocytes and dendritic cells in HIV-infected individuals that included increased levels of IL-12, TNF-α, and IFN-α. While this pro-inflammatory phenotype was evident in certain cell types (CD11b+ activated monocytes, myeloid dendritic cells) for HIV-negative older adults, the most striking differences were associated with HIV-infection. Our previous studies of Mincle, another C-type lectin receptor family member recognizing carbohydrate motifs including M. tuberculosis cord factor, also showed an increase in cytokine production in monocytes with age and HIV infection [32]; thus HIV and age appear to enhance cytokine production downstream of C-type lectin signaling and represent an additional factor that could contribute to age-related chronic inflammation.

Our analyses of RNA-seq experiments on purified CD14+ CD16+ monocytes from young and older adults with and without HIV infection were notable for a distinct inflammatory signature in the HIV-older population. Gene expression from several pro-inflammatory pathways was enhanced at baseline in this group, such as TNF-α signaling (and in this regard, Dectin-1-dependent TNF-α production in inflammatory monocytes was increased with HIV infection in both young and older adults (Figure 1)). Increased GSEA enrichment of the IL-2/STAT5 signaling pathways was also noted in the HIV-positive Older cohorts (also seen in HIV-negative Older cohort). The IL-2/STAT5 signaling pathway although traditionally thought to be a T cell pathway, has been reported in monocytes where it may play a role in anti-fungal immunity [33]. Additionally, there was also increased baseline expression of IL-19/IL-24 (Supplementary Figure 4A), and the KEGG complement/coagulation pathways (Supplementary Figure 4C). The link between Dectin-1 and coagulation factors is unclear, but both F3 (tissue factor and PLAUR (plasminogen activator) were hub genes only for the HIV-Older group (Figure 5A). Because increased production of clotting factors is also associated with age-associated chronic inflammation [13], our results suggest CLR signaling as an additional contributing factor. Finally, increased Reactive Oxygen species (ROS) pathway signaling was noted in the Older HIV-negative and HIV-positive cohorts (with stimulation) (Figure 4). However, baseline analysis only showed increased enrichment of the ROS signaling in the HIV-Older cohort (Supplementary Figure 7). Overall, HIV-infected older adults demonstrated unique gene expression with Dectin-1 stimulation when compared to the other cohorts.

Studies in the early years of the HIV pandemic reported higher plasma levels of IFN-α in HIV-infected individuals in both acute and chronic infection [34] that was strongly associated with the immune activation seen with chronic HIV-infection and is thought to contribute to pathogenesis. Our studies indicate that Dectin-1 signaling, particularly in HIV-infected individuals, may contribute to this dysregulated IFN production. Notably, both mDCs and pDCs showed an HIV-associated increase in Dectin-1-induced IFN-α production in young and older adults (Figure 1C, 1D). This increase in IFN-α production also paralleled the increased Dectin-1 surface expression in both mDCs and pDCs noted with HIV-infection (Figure 2E). We also found increased enrichment of Dectin-1-dependent IFN-α and IFN-γ pathway expression only in the HIV-Older group, in contrast with downregulated IFNα and IFN-γ responses in all other cohorts (Figure 4 and Supplementary Figure 5). In this regard, we found an age-associated decrease in basal IFN-α and IFN-γ pathway expression that may contribute to enhanced Dectin-1-dependent IFN signaling in HIV-Older adults. A post-hoc analysis of monocytes (done after RNA-seq results) did demonstrate that both IFN-α and IFN-γ are produced in monocytes in response to Dectin-1 stimulation (Supplementary Figure 11). Interestingly, we found both IFN-α and IFN-γ levels at baseline to be increased in the HIV-older cohort when compared to HIV-negative young and older adults. Dectin-1 stimulation showed comparable levels of IFN-α production amongst the cohorts, with a significant difference in IFN-γ production where HIV-older adults demonstrated decreased IFN-γ production when compared to HIV-negative young adults. Although controversial, IFN-γ production has been definitely noted in antigen presenting cells (myeloid cells such as monocytes) in prior studies [35]. IFN-α has been reported to downregulate the production of IFN-γ by antigen presenting cells [36]—in this case the strong IFN-α signature seen in the HIV-older cohort could contribute to the decreased IFN-γ production seen in HIV-older adults in response to Dectin-1 stimulation. However, the already elevated levels of IFN-γ seen at baseline in HIV-older adults points to dysregulation of the IFN pathways in the setting of HIV-aging. While traditionally associated with antiviral immunity, there is accumulating evidence that interferon responses (Type I and Type III) also modulate the antifungal immune response [37–39]. Production of IFN-γ has not been previously reported in myeloid cells in response to Dectin-1 stimulation—although, treating myeloid cells with IFN-γ prior to a fungal challenge was associated with enhanced inflammation [40, 41]. Although, type I IFN production may play an important role in the antifungal response, it may also result in dysregulated inflammatory responses in monocytes that lead to worsened outcomes from fungal infection [42] as can be seen in older adults with HIV infection. Additionally, the increased and persistent production of IFNs via Dectin-1 stimulation may also be contributing to the pro-inflammatory environment that is seen in HIV-infection and Aging.

Analysis of our RNA-seq data revealed the innate immune receptor Triggering receptor in myeloid cells-1 (TREM-1) as a significant upstream regulator in all cohorts (Figure 5B). There are limited studies demonstrating a relationship between TREM-1 and Dectin-1 in fungal pathogenesis, although expression of TREM-1 and Dectin-1 both increased with severity and progression of an *Aspergillus* keratitis mouse model [21], with inhibition of both TREM-1 and Dectin-1 resulting in protection from fungal disease. TREM-1 is a receptor expressed on neutrophils and monocytes that modulates the inflammatory response [43, 44], and is upregulated in sepsis. Although, the specific agonist for TREM-1 remains unknown, the receptor is known to interact with CARD9 in a complex with Bcl-10 and MALT-1, hinting at a possible interaction with C-type lectin receptors [43]. The significant upregulation of TREM-1 noted in our study also alludes to a physical interaction between TREM-1 and Dectin-1.

Other upstream regulators that emerged included the cytokines TNF-α, IL-1β that were significant in all cohorts, and IFNs that were prominent negative regulators in the HIV-Young cohort. Other important regulators that emerged was the NF-κB complex, and the MAP kinases ERK, and p38 pathways which have been reported as important components of the Dectin-1 signaling [1, 45]. In fact, ERK and p38 were shown to regulate zymosan induced IL-10 production in a mouse model [45]. IL-10 was robustly produced by all cohorts in our study in response to Dectin-1 stimulation. Dectin-1 stimulation also increased expression of the anti-inflammatory cytokines IL-19 and IL-24 in the CD14+CD16+ monocytes in most cohorts, with the HIV-Older cohort already increased at baseline (Supplementary Figure 4A).

Other notable differences should be pointed out. The HIV-Older group showed decreased gene expression of IL-7R gene expression with Dectin-1 stimulation when compared to all other cohorts (Supplementary Figure 4A). Expression of IL-7R in monocytes has been reported in the setting of LPS exposure and its role remains to be fully defined [46]. Decreased expression of IL-7R in monocytes has not been reported in the setting of HIV and aging. However, decreases in IL-7R expression and impaired IL-7 signaling in T cells have been reported in the setting of both aging [47] and HIV-infection [48]. Another gene of note was IL-1β which was noted to be increased at baseline in all cohorts except for the HIV-Older cohort (Supplementary Figure 4A). Dectin-1 stimulation led to an upregulation of IL1-β, in all cohorts (Supplementary Figure 4A–4D), although higher in all groups compared to the HIV Older group. Dectin-1 is a known inducer of the inflammasome [49, 50]. Interestingly, IL-1β emerged as a significant upstream regulator of Dectin-1 signaling in all cohorts, in addition to an important hub gene (most cohorts) as noted in Figure 5A, 5B. Further study of the Dectin-1 induced inflammasome is needed in the setting of HIV-infection and aging.

Overall, Dectin-1 stimulation led to the upregulation of several metabolic pathways, including mTORC1 signaling, the hypoxia pathway, and P13K-Akt signaling. Upregulation of the MTORC1 signaling and the Hypoxia pathway was noted all cohorts (Figure 4). Baseline GSEA pathway analysis also demonstrated increased MTORC1 pathway enrichment in HIV-Older adults which was absent in all other cohorts. (Supplementary Figure 7). KEGG gene enrichment analysis showed upregulation of PI3K-Akt pathway in all groups except the HIV Old cohort (Supplementary Figure 3), although was also noted to be upregulated at baseline (Supplementary Figure 6). Activation of the Akt/mTOR/HIFβ–α pathway is thought to be the metabolic basis for trained immunity in Monocytes trained with β–glucan [51], and trained immunity is likely playing a role in the anti-fungal response in humans. Our findings show that trained immunity may also be contributing to the sustained pro-inflammatory phenotype seen in the setting of HIV and aging, a finding which has also been noted in other studies [52]. Furthermore, Dectin-1 stimulation also led to a strong upregulation of the unfolded protein response in all cohorts (Figure 4). There is evidence that the unfolded protein response may be important for proper innate immune function and may be necessary for the proper functioning of dendritic cells [53].

Finally, Dectin-1 led to the upregulation of several differentiation signatures (Figure 6) alluding to the possibility that Dectin-1 stimulation may lead to the differentiation of monocytes into both dendritic cells and macrophages. Specifically, Dectin-1 induced transcriptomic changes in monocytes suggest the differentiation of CD14+CD16+ monocytes mainly to monocyte-derived dendritic cells—changes that are noted in all the cohorts (Figure 6B). However, the transcriptomic profile of the stimulated inflammatory monocytes of HIV-positive older adults also revealed a distinct gene signature that demonstrated an activated DC signature, as well as a more inflammatory M1 macrophage signature. A prominent activated DC signature was also seen in Older HIV-negative adults with co-morbidities (a smaller upregulation was noted in Young HIV-negative adults with co-morbidities). Young HIV-negative adults seemed to skew towards a non-activated dendritic cell signature. Activation of the PPAR-γ pathway has been shown to inhibit Dectin-1 induced dendritic cell activation in human PBMCs [54] from healthy volunteers. Interestingly, KEGG DEG analysis did not show upregulation of the PPAR signaling pathway in the HIV-Older and Older HIV-negative group as compared to young adults where it was upregulated. However, baseline pathway analysis also showed upregulation of PPAR signaling at baseline in the HIV Older cohort—alluding to more complex signaling in the setting of HIV and aging. Overall, Dectin-1 signaling in the setting of HIV and aging was associated with a more inflammatory activated DC signature and M1 macrophage signature. Additionally, the output of inflammatory mediators brought on by Dectin-1 simulation could promote different T cell responses. In fact, Dectin-1 stimulation in dendritic cells has been shown to promote Th17, Th1, Th2 and cytotoxic CD8 responses [55]. Future experiments will be needed to discern how HIV and aging affect these downstream T cell responses from Dectin-1 stimulation.

Other roles for Dectin-1 have emerged which may also be playing a role in the setting of HIV-infection and aging. There is accumulating evidence that Dectin-1 recognizes endogenous ligands such as annexins, and vimentins from dying cells [9]. Vimentin when bound to Dectin-1 in atherosclerotic plaques induces ROS production in human monocytes [56]. Dectin-1 activation induced by vimentin promoted obesity and insulin resistance in a diet-induced obesity mouse model. This same study showed increased Dectin-1 expression in human adipose tissue samples of obese individuals when compared to lean subjects [57]. Interestingly, a recent study of caloric restriction in humans showed a downregulation of Dectin-1 signaling in RNA seq studies of subcutaneous adipose tissue after two years of 14% caloric restriction [58]. In our study, Dectin-1 surface expression in activated monocytes was examined in the context of a multivariable regression model (Table 2). Within our fully adjusted model, the co-variates of HIV-infection, recreational drug use, number of co-morbidities, and percent lifespan with HIV all proved to be significant. The significance of number of co-morbidities in the model of Dectin-1 surface expression suggests that factors unrelated to fungi are important for Dectin-1 surface expression and need to be further explored. Therefore, the stimulation of Dectin-1 by endogenous ligands could certainly be contributing to both age and HIV-associated chronic inflammation as seen in our study.

In addition to endogenous ligands, circulating fungal ligands could also be contributing to these notable age and HIV-associated differences. Elevated levels of circulating β-D-glucan levels have been noted in several studies in the setting of chronic HIV-infection that persist despite long term Antiretroviral therapy (ART) and is thought to be secondary to microbial translocation [59, 60]. Previous studies have shown that elevated levels of β-D-glucan was associated with markers of immune activation (soluble CD14, HLA-DR+ CD4/CD8 T cells, and IL-6/IL-8 levels) and decreased Dectin-1 surface expression on monocytes from individuals with HIV infection, compared to HIV-negative controls [59]. HIV-positive and negative individuals in this study were not segregated into age groups, and so it is not clear if the general HIV-associated increase in Dectin-1 expression we observed would have been found; in addition, there were methodological differences between our studies, such as the analysis of freshly isolated PBMCs (this study), versus cryopreserved samples. Our findings showed increased Dectin-1 surface protein expression with both increased age and HIV-infection. Evaluation of Dectin-1 (Clec7a expression) gene expression (Supplementary Figure 10) showed an opposite trend of decreased gene expression levels in the HIV-Older cohort and increased levels in the young HIV-negative cohort. These findings suggest a mechanism that is regulated post-transcriptionally in the HIV-Older cohort that needs to be further investigated. A recent paper that examined monocytes from people living with HIV found a strong correlation between serum levels of β-D-glucans and production of IL-1β in general (although specific stimulation with Candida albicans led to decreased cytokine production in this study) and suggested that the robust production of cytokines by monocytes in HIV-infection was linked to trained immunity in monocytes [52]. Microbial translocation is something that is also likely occurring with increased age [61] as increased levels of surrogate markers of microbial translocation have been noted in older adults. Therefore, our findings of a unique pro-inflammatory signature in the setting of HIV and aging could be secondary to elevated levels of circulating of β-D-glucans that have been noted in the setting of chronic HIV-infection [13].

Overall, this study demonstrates that age, HIV-infection and co-morbidities can alter the individual immune response. In particular our study showed a unique immune signature in the setting of both HIV and aging in response to Dectin-1 stimulation. As our HIV-positive population ages these immune differences will need to be considered for future vaccines and therapeutics. Dectin-1 agonists such as β-D-Glucans have been proposed to be possible adjuvants for vaccines as they induce epigenetic changes associated with trained immunity [62, 63]—and so could represent a useful immune response. However, Dectin-1 induced inflammation possibly mediated by trained immunity could also contribute to the immune activation or chronic inflammation that is seen in the HIV-infected population. Therefore, Dectin-1 induced inflammation could represent a double-edged sword in the HIV/Aging population. Further studies are needed to dissect these mechanisms. At the very least we need to consider the HIV/Aging population as a unique population in clinical studies given their unique inflammatory profile.

## MATERIALS AND METHODS

### Clinical study design and recruitment of participants

We recruited a total of 81 HIV-negative and HIV-positive participants, divided between young (21-35) and older (≥ 60 years) adults (Note: the age range for the Dendritic cell experiments was extended to 21-40 and ≥ 50 years). Subjects were recruited from the Yale Health Services, Yale Primary Care Center (PCC), and the Nathan Smith HIV Clinic. Participants were evaluated for clinical characteristics by chart review and self-report for demographic information, medications, CD4 count, HIV viral load, and comorbid conditions. Participants were excluded for the following reasons: an acute infection or antibiotic use within two weeks of recruitment, pregnancy, history of current cancer, history of stem cell, bone marrow or solid organ transplant, cirrhosis of the liver, kidney disease requiring dialysis, immunodeficiency other than HIV, and active Hepatitis B or C infection. Of note, in the RNA seq experiments we do note the following terms Young, Young*, Older*, and HIV-young and HIV-Older. Young* and Older* refer to subjects with increased co-morbidities (counted number of co-morbid conditions) as compared to the Young and Older groups. In our initial experiments (flow cytometry) we noted that our HIV+ cohorts had increased co-morbidities compared to our HIV-negative cohorts. Therefore, for subsequent recruitments, including the RNA seq experiments we aimed to recruit HIV-negative adults that had more matched co-morbid conditions.

### Sample preparation

Peripheral blood mononuclear cells (PBMCs) were isolated from heparinized blood using Ficoll gradient centrifugation (Histopaque Sigma) as previously described [18, 64]. Freshly isolated PBMCs were stimulated with 10 μg/ml of whole glucan particles (WGP Dispersable or 1,3/1,6-β-glucan) (InvivoGen, tlr-wgp) which is a specific Dectin-1 agonist that lacks TLR stimulating activity—for 18 hours (for Monocyte experiments), and 12 hours (for dendritic cell experiments) in RPMI medium supplemented with 10% fetal bovine serum and Penicillin/Streptomycin/L-Glutamine at 37° C. WGP (Soluble, or 1,3/1,6-β-glucan) (Invivogen tlr-wgps) was used as a negative control. WGP soluble particles can bind to the Dectin-1 receptor without activating it. Baseline samples were completely unstimulated. Brefeldin-A (GolgiPlug, BD Biosciences) was added for the last 6 hours of stimulation. Cells were then surface stained with anti-CD14-PE-CF594 (3G8, BD-Biosciences), anti-CD16-PE-Cy7 (3G8, BD-Biosciences), and anti-CD11b-APC-Cy7 (ICRF44, BD-Biosciences) to identify monocytes and separate them into activate, inflammatory, classical and non-classical subsets. Dendritic cells were identified by staining for lineage markers (CD3, CD14, CD16, CD19, PE-TXR, BD Biosciences) in a general dump gate. Lineage negative cells that were also anti-HLA DR+-APC-Cy7) (LN3, eBioscience) (Lin-/HLA DR+) were then stained for anti-CD11c-PE-Cy7 (3.9, eBioscience), and anti-CD123-APC (7G3, BD Biosciences) to separate dendritic cells into myeloid dendritic cells (CD11c+, CD123-), and plasmacytoid cells (CD11c-, CD123+). Cells were fixed in Cytofix buffer (BD-Biosciences) and stored at -80° C in freezing medium until analyzed using flow cytometry. On the day of analysis cells were thawed, washed, and permeabilized with Cytofix/Cytoperm (BD-Biosciences) and Perm/Wash buffer (BD-Biosciences) for intracellular cytokine staining with anti-interleukin-10 (IL-10) Pacific Blue (JES3-9d7, eBioscience), anti-interleukin-12 p70 (IL-12) PE (20C2, BD Biosciences), anti-interleukin-6 (IL-6) fluorescein isothiocyanate (MQ2-13A5, eBioscience), and anti-tumor necrosis factor-α (TNF-α) Alexafluor 700 (MAB11, BD Biosciences) for monocyte staining. Dendritic cells intracellular staining used the following antibodies: anti-IFN-α-FITC (pbl interferon source, 21112-3), anti-interleukin-12 p70 (IL-12) PE (20C2, BD -Biosciences), anti-IL-6-Alexaflour 700 (MQ2-13A5, eBioscience), anti-tumor necrosis factor-α (TNF-α)-Pacific blue (Mab11, eBioscience). Samples were run using a Fortessa LSRII flow cytometer (Becton Dickinson) and analyzed using FlowJo software (FlowJo, LLC). A general gating strategy is shown in Supplementary Figure 1. Cells were also analyzed for Dectin-1 surface expression (anti-Dectin-1, 15E2, eBioscience) by flow cytometry.

### RNA seq studies/monocyte sorting and whole glucan particle stimulation

Isolated PBMCs were washed and stained for CD14 PE-TXR and CD16 PE-Cy7 for 30 mins at 4° C in the dark. The cells were then filtered and resuspended in FACS buffer and sorted using a BD FACS Aria II. The sorted CD14+CD16+ monocytes were transferred into a round bottom 96 well plate and stimulated with whole glucan particle (10mg/ml) for 18 hours at 37° C in a 5% CO2 humidified environment. The cells were further processed for RNA isolation by using Qiagen RNeasy kit as per the manufacturer’s instructions.

### RNA-seq

The library preparation and sequencing were performed in Yale Stem Center Genomics Core Facility by Dr. Mei Zhong. The raw FASTQ files were executed for adaptor trimming and quality trimming using Cutadapt v1.18 [65]. The quality control of the raw and trimmed FASTQ files were performed using FASTQC v0.11.7 [66]. The trimmed files were aligned to human genome reference (GRCh38.primary assembly) by using STAR/ 2.7.3a-foss-2018b with parameters –runThreadN 12 --outFilterMultimapNmax 1 --outFilterMismatchNmax 999 --outFilterMismatchNoverLmax 0.02 --alignIntronMin 20 --alignIntronMax 1000000 --alignMatesGapMax 1000000 --sjdbOverhang 100 --outSAMtype BAM SortedByCoordinate [67]. The BAM files were further sorted using SAMtools/1.9-foss-2018b and raw counts were obtained using Subread/2.0.0-GCC-7.3.0-2.30 featureCounts package and gencode v29 annotation.

### Differential gene expression

The counts from protein-coding genes, ncRNA and lincRNA were analyzed for differential gene expression in WGP Stimulated vs. Unstimulated samples using DESeq2 in R 4.0.3. Briefly, the counts were pre-filtered to remove low read (<10). For batch correction, co-variate batch was included in the design (design=~batch+condition). The genes with a log fold change cut of 1.2 and FDR of < 0.1 were considered differentially expressed genes (DEGs). The normalized counts were also obtained using DESeq2 for further analysis. The reads obtained from Y-chromosome were not included in the analysis.

### Gene set enrichment analysis (GSEA)

Gene set enrichment analysis was carried out on the normalized counts using Broad Institute GSEA v 4.1.0 [68]. The Hallmark, C2 REACTOME, and C7 ImmuneSigDB gene sets v7.4 from Molecular signature database (MSigDB) were used for the analysis [69]. The GSEA was run at 1000 permutation. The gene set with FDR of less than 5% was considered significant.

### Functional enrichment and protein-protein interaction analysis

The upregulated and downregulated DEGs were analyzed for the functional enrichment analysis by using Enrichr web server [70, 71]. The enrichment was performed for GO molecular function and biological processes and for Kyoto Encyclopedia of Genes and Genomes (KEGG). The significance of the enrichment was calculated using Benjamini-Hochberg FDR and terms with FDR of less than 1% were considered significant. The prediction of protein-protein interaction network was made for DEGs using searching tool for the retrieval of interacting genes (STRING) database [72]. The minimum interaction score of 0.4 was used for interaction prediction. The networks of highly interacting nodes were further visualized using Cytoscape v 3.9.0 [73]. The hub genes were identified based on the degree of node using Cytohubba plugin [74].

### Upstream regulator analysis

The Qiagen Ingenuity Pathway Analysis (IPA) upstream regulator analysis [75] identifies the genes that are upstream regulators and thus regulate downstream target genes. The prediction of upstream regulators was performed for the DEGs. The upstream regulators were identified based on the activation or inhibitions z-score. The z-scores with the cutoff of 2 and p-value <0.05 were considered significant.

### Statistical analyses for flow cytometry studies

To assess overall cytokine expression as a function of specific participant characteristics, a series of non-parametric tests of medians were computed for each cytokine between specific subsets of the patient group (Older vs. Younger, among HIV-positive; Older vs. Younger, among HIV-negative; HIV-positive vs. HIV-negative, among Older patients; and HIV-positive vs. HIV-negative, among Younger patients). Results were FDR corrected for the set of cytokines assess within each cell type (Inflammatory monocyte, activated monocyte, classical monocyte, non-classical monocyte, and myeloid dendritic cell, plasmacytoid dendritic cells). To further explore the relationship of HIV status and age within specific cytokine expressions, parametric multivariable regression models were run for Dectin-1. First, we ran an unadjusted model that included HIV status (HIV-positive/HIV-negative), age (Older/Younger) and an HIV by age interaction. Second, we included an adjusted model that also included potentially relevant covariates. These included indicators for the use of drugs, the presence of a fungal infection, a four-level categorical variable indexing the number of comorbid conditions, and the percent of the individual’s lifespan and they have lived with HIV. Finally, we used stepwise backwards selection on the fully adjusted model to derive a parsimoniously specified model. All statistics were carried out using SAS software. Copyright, SAS Institute Inc. SAS and all other SAS Institute Inc. product or service names are registered trademarks or trademarks of SAS Institute Inc., Cary, NC, USA.

### Study approval

This study was approved by the Human Research Protection Program of the Yale School of Medicine. Informed consent was obtained from all subjects prior to participation.

## Supplementary Material

Supplementary Figures

Supplementary Table 1

Supplementary Tables 2-4

Supplementary Table 5
