# Supplementary material for: Dectin-1 stimulation promotes a distinct inflammatory signature in the setting of HIV-infection and aging
Source: Aging (Albany NY). 2023 Aug 21;15(16):7866–908. doi: 10.18632/aging.204927 (PMC10497004; doi:10.18632/aging.204927)
Supplement: Supplementary Figures [file aging-15-204927-s001.pdf]

SUPPLEMENTARY FIGURES

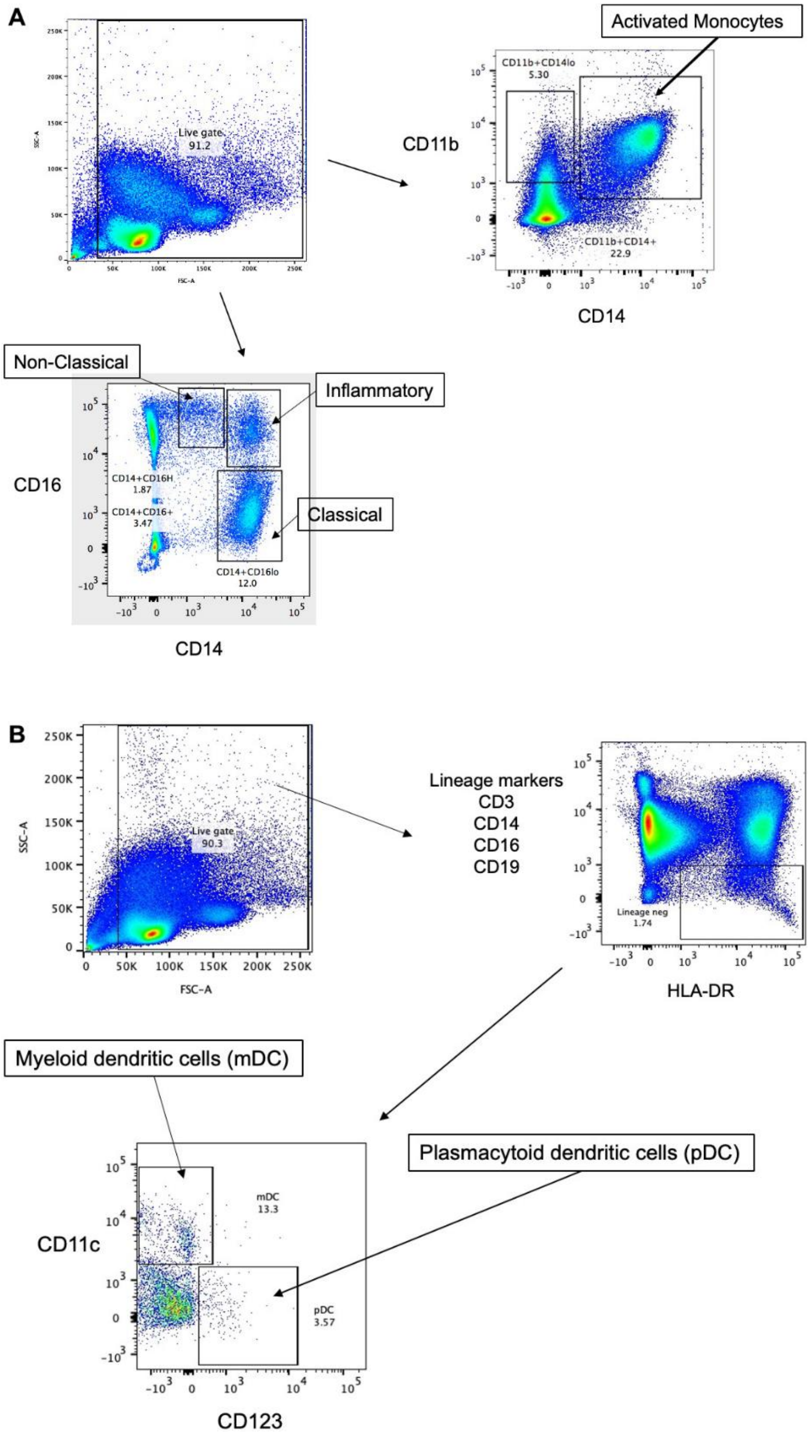

C

	mDC (%)	MFI IFN-α	MFI TNF-α
Baseline	11.4	59.2	-203
Stimulation	26.3	112	1828

	pDC (%)	MFI IFN-α	MFI TNF-α
Baseline	11.4	49.4	-241
Stimulation	26.3	85.5	352

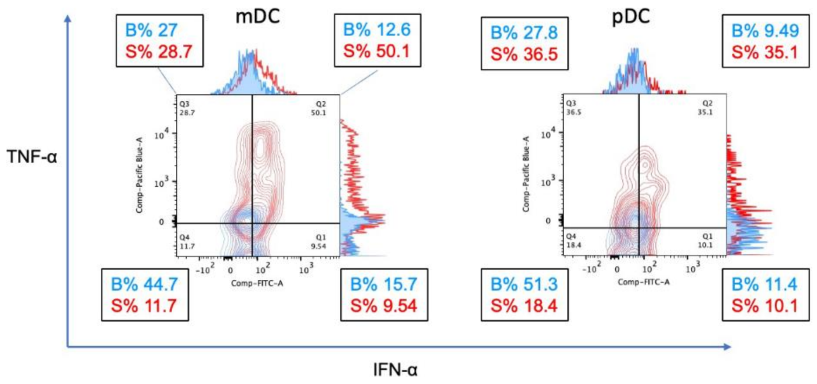

Supplementary Figure 1. Flow cytometry gating strategy and cytokine analysis. (**A**) Monocyte gating strategy. PBMCs were surface stained with anti-CD14-PE-CF594 (3G8, BD-Biosciences), anti-CD16-PE-Cy7 (3G8, BD-Biosciences), and anti-CD11b-APC-Cy7 (ICRF44, BD-Biosciences) to identify monocytes and separate them into activated (CD11b+CD14+), Inflammatory (CD14+CD16+), Classical (CD14+CD16lo), and non-classical monocytes (CD14+CD16H). Figure 1A shows the gating strategy for monocytes. All gating started with a live gate. Compensations were done for every experiment, along with isotype controls. Flow cytometry was analyzed using FloJo (LLC). (**B**) Dendritic cell gating strategy. Dendritic cells were identified by staining for lineage markers (CD3, CD14, CD16, CD19, PE-TXR, BD Biosciences) in a general dump gate. Lineage negative cells that were also anti-HLA DR+-APC-Cy7) (LN3, ebiosciences) (Lin-/HLA DR+) were then stained for anti-CD11c-PE-Cy7 (3.9, ebioscience), and anti-CD123-APC (7G3, BD Biosciences) to separate dendritic cells into myeloid dendritic cells (CD11c+, CD123-), and plasmacytoid cells (CD11c-, CD123+). All gating started with a live gate. Compensations were done for every experiment, along with isotype controls. Flow cytometry was analyzed using FloJo (LLC). (**C**) Cytokine gating strategy. Specific cell populations (shown here are mDC and pDC populations) were then gated on to evaluate cytokines using quadrant analysis as shown in Figure 1C. Percent (%) change represents the percentage of cytokine (TNF-a, IFN-a) positive dendritic cells (shown here are the gating for both mDCs and pDCs) that is positive for a cytokine when the baseline is subtracted from the stimulation. Quadrants were added up to get the overall % change. For example for the mDC population TNF-α we did the following calculation: Quadrant 3 + Quadrant 2 = % positive for TNF-α (27% + 12.6% = 39.6 % positive for TNF-α at Baseline, 28.7 + 50.1% = 78.8 % positive for TNF-α with Stimulation). Percent change (%) of TNF-α with stimulation would be 39.2% (78.8% - 39.6%). Mean Fluorescence Intensity was also reviewed for all cytokines to make sure it correlated with dot plots and shown here with histograms and table. B% (Baseline percent positive), S% (Stimulation percent positive). Corresponding MFI values are shown in table, alongside % of mDCs and pDCs (overall of parent).

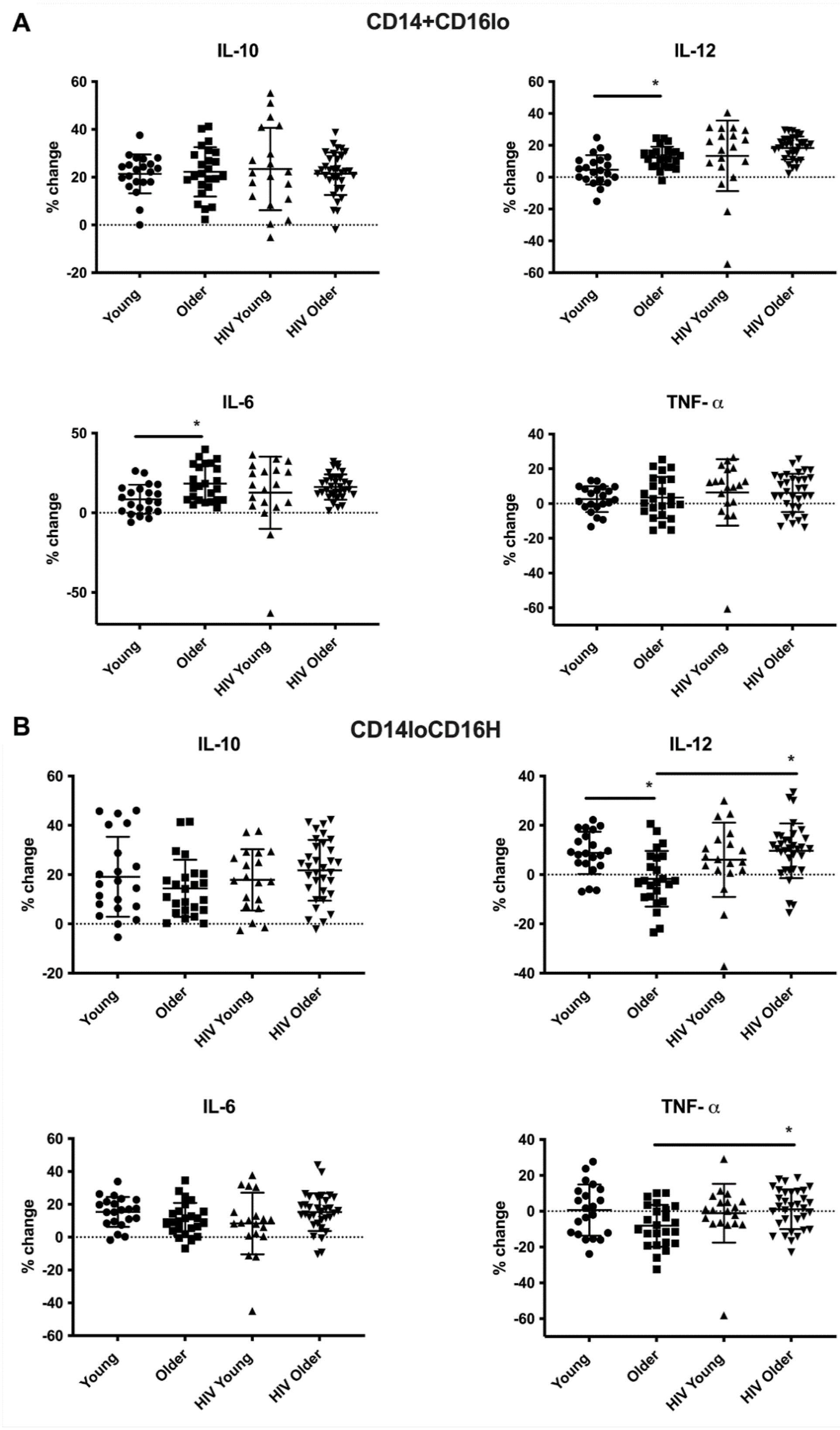

Supplementary Figure 2. Effects of age and HIV-infection on Dectin-1 induced cytokine production in classical and non-classical monocytes. (**A**) Total cytokine production in classical monocytes (CD14+CD16lo). Young vs. Older, IL-12 (p= 0.0281), IL-6 (p= 0.0281). All other comparisons were not significant. (**B**) Total cytokine production in nonclassical monocytes (CD14lo, CD16H). Young vs. Older, IL-12 (p= 0.044), Older vs. HIV-Older, IL-12 (p=0.0488), TNF-α (p= 0.0488). All other comparisons were not significant.

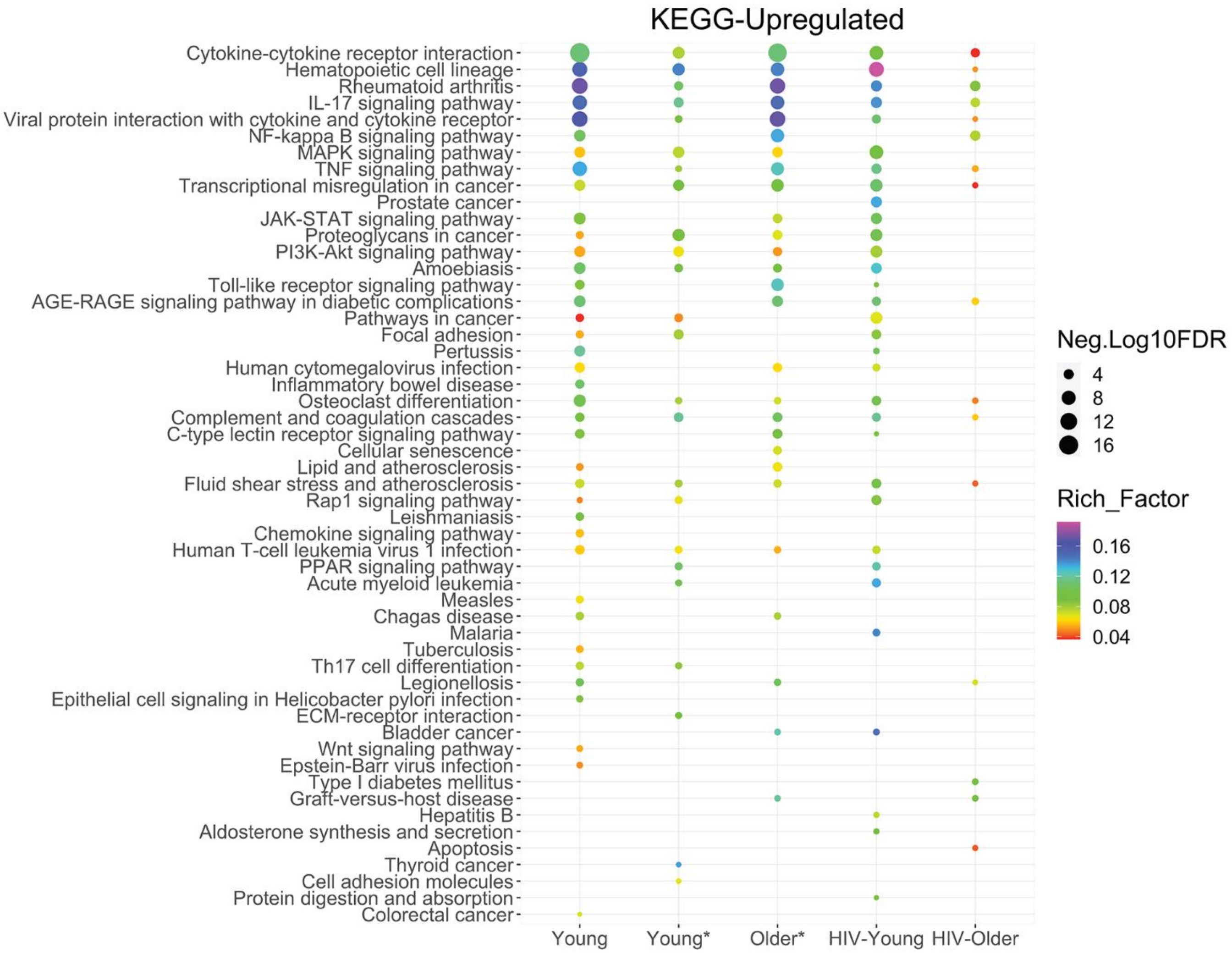

Supplementary Figure 3. KEGG pathway analysis of Dectin-1 stimulated monocytes. KEGG analysis was performed for Dectin-1 stimulated Inflammatory monocytes of all cohorts using Enrichr. Dot plots represent significant KEGG pathways with FDR of < 1%. The size of the node represents enrichment factor which is defined by overlap of the input to the gene set, color of node represents -Log10(FDR).

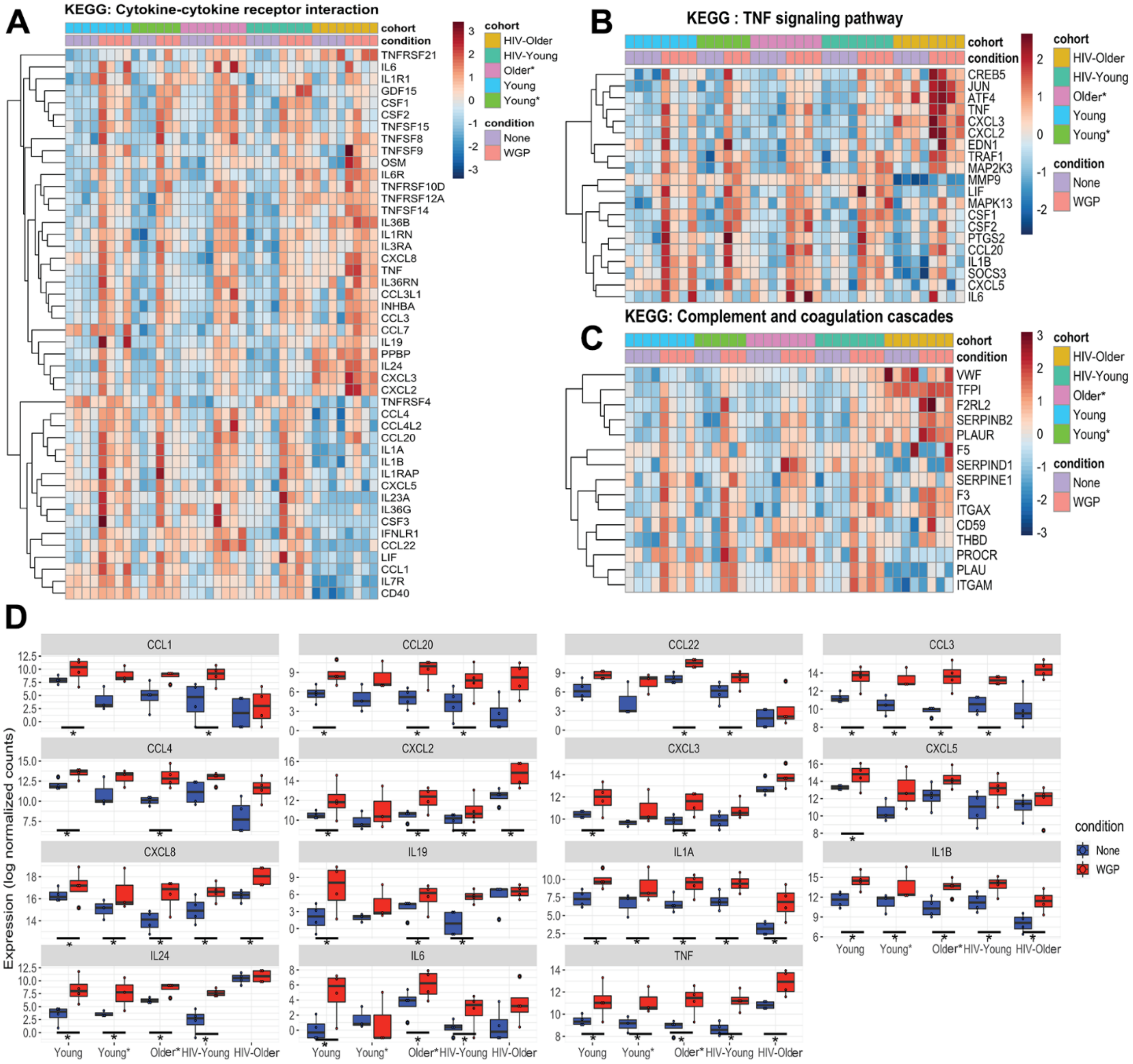

Supplementary Figure 4. Dectin-1 stimulation promotes TNF-α signaling, and induction of the coagulation cascade in the HIV-Older cohort. Heatmaps showing KEGG gene sets of Inflammatory Response, TNF-α and coagulation signaling. (**A**) Cytokine-cytokine receptor interaction and (**B**) TNF signaling pathways and (**C**) Complement and coagulation cascades. (**D**) Boxplots represent log normalized counts of cytokines and chemokines in unstimulated and WGP stimulated Inflammatory monocytes. The symbol * represents significant differentially expressed genes with fold cutoff of 1.2, and q value < 0.1.

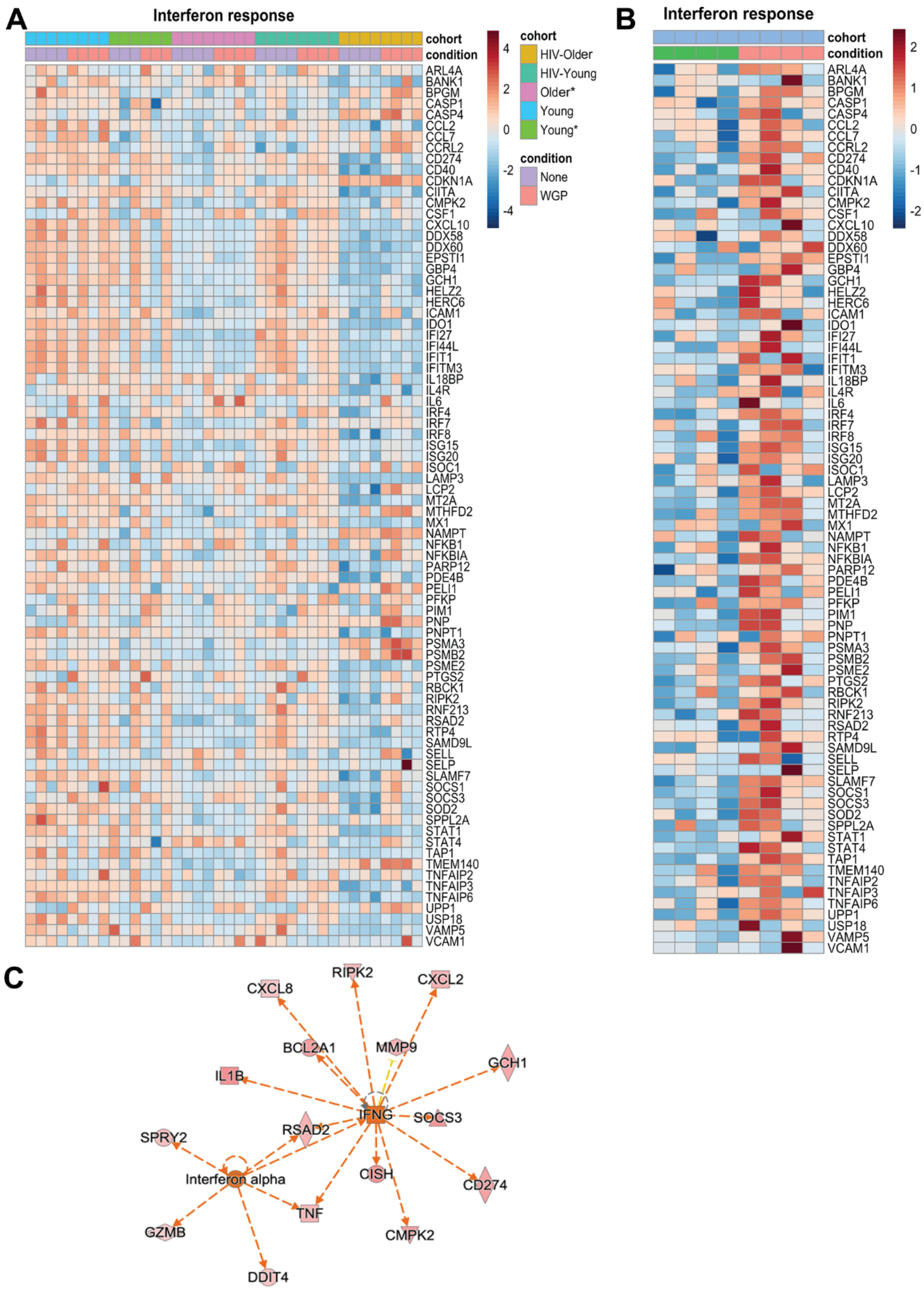

Supplementary Figure 5. IFN-α/γ response is enriched in HIV-infected older adults with Dectin-1 stimulation. (**A**) Heat map showing the expression profile of the gene sets that showed enrichment for Hallmark Interferon alpha and gamma response in Dectin-1 stimulated monocytes of HIV-older adults compared to other groups. (**B**) Heatmap focusing on the response in HIV older adults, showing expression profile of the gene sets that showed enrichment for Hallmark Interferon alpha and gamma response. (**C**) IPA upstream analysis shows IFN-γ and IFN-α as an upstream regulators and their target genes in Dectin-1 stimulated monocytes of HIV-older adults.

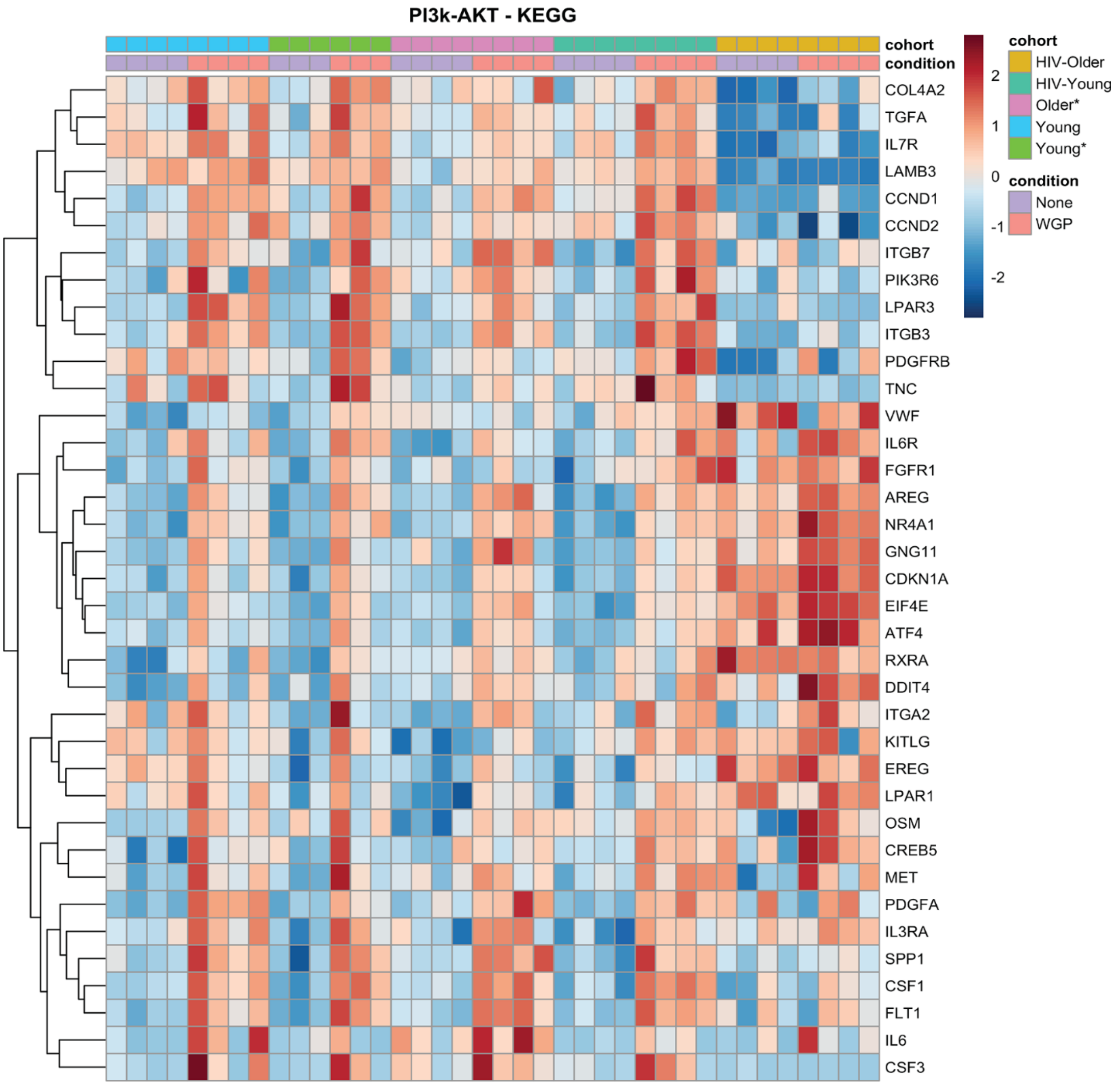

Supplementary Figure 6. PI3K-Akt pathway is upregulated at baseline in the HIV-older cohort. Heatmap showing expression profile of genes significantly enriched for PI3k-Akt pathway during KEGG analysis. The heatmap was constructed using Pheatmap. The transcripts were normalized using variance stabilizing transformation function. The color represents relative expression of transcripts that covary across cohorts and condition.

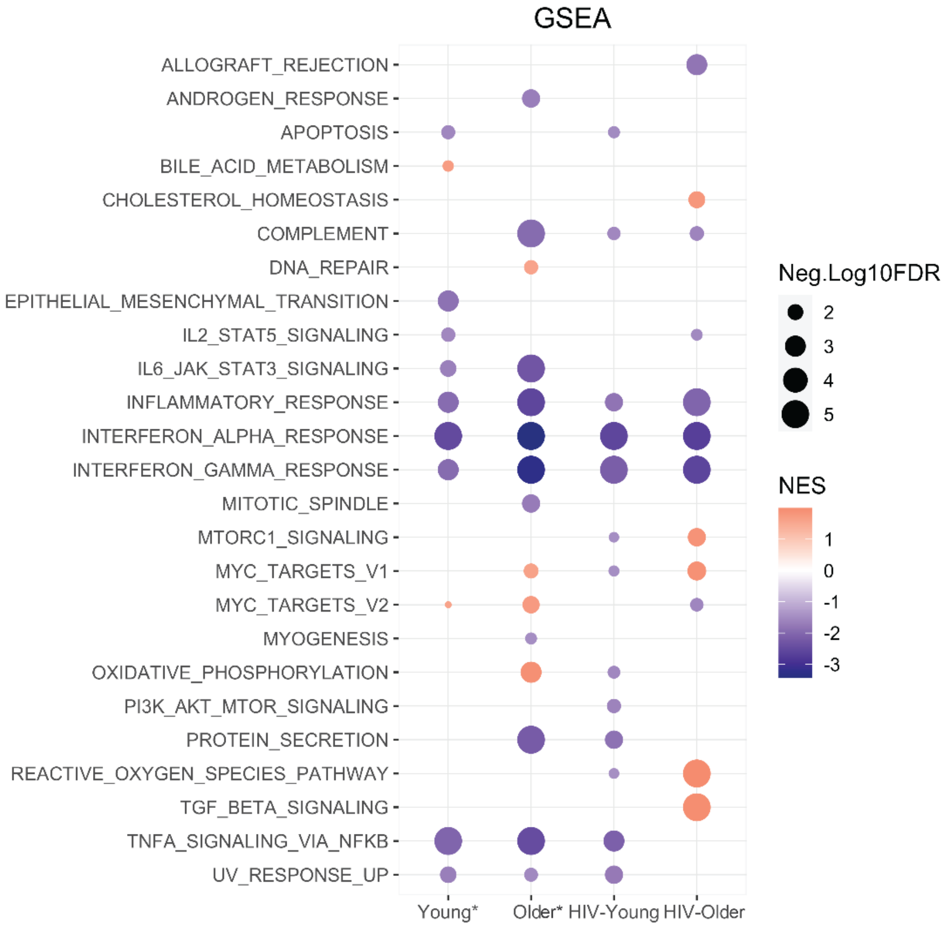

Supplementary Figure 7. GSEA/Hallmark baseline analysis. Baseline Gene Set Enrichment Analysis was performed using the Hallmark gene sets for each cohort. The dot graph represents the significant Hallmark pathways identified in the isolated monocytes of a respective cohort when compared to monocytes of healthy young individuals. The pathways with FDR of <5% were considered significant. For graphical representation, FDR values with 0 were adjusted to 0.00001. The size of the node represents -Log10(FDR) and color of node represents normalized enrichment score. NES = Normalized enrichment scores.

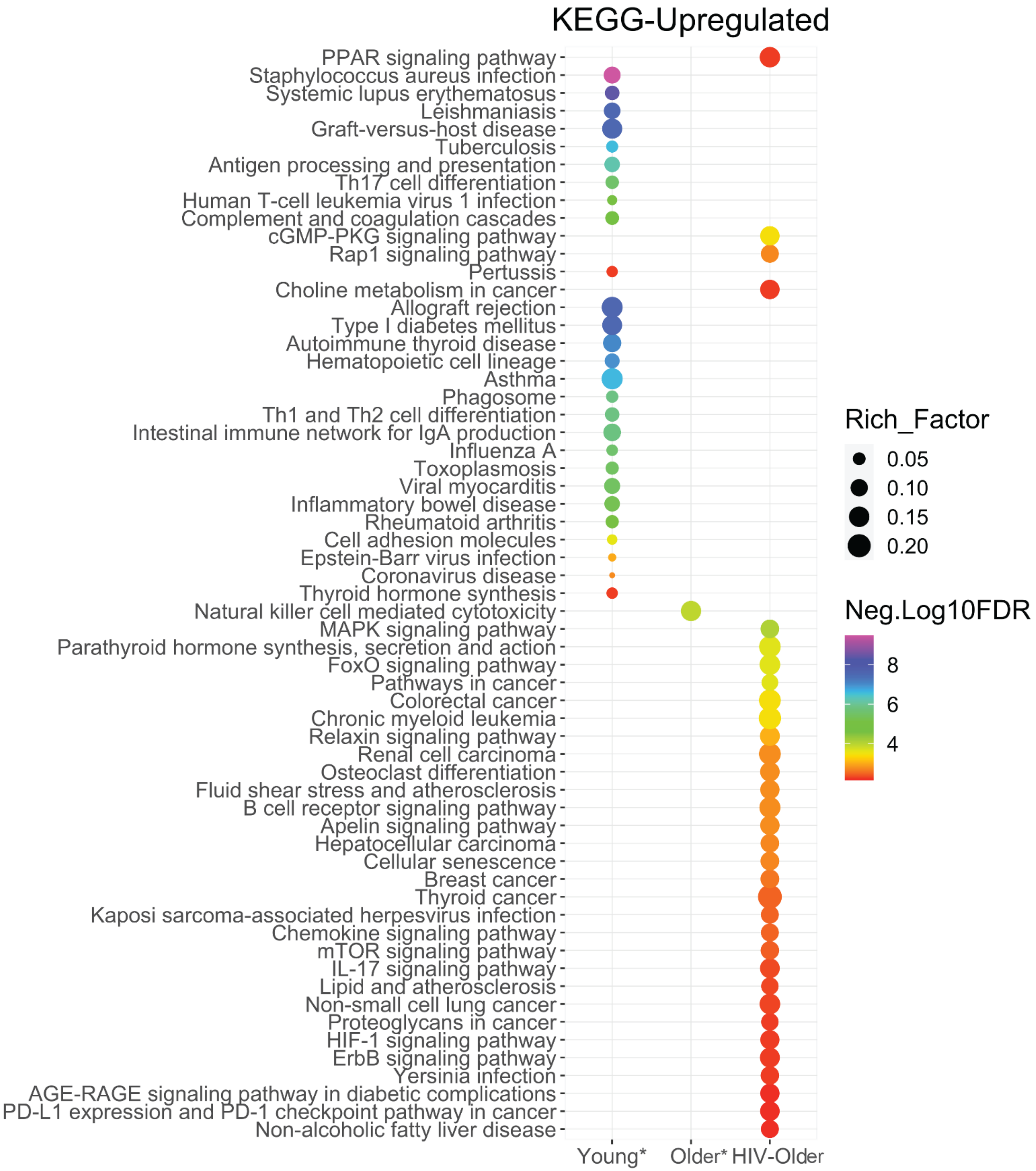

Supplementary Figure 8. KEGG baseline pathway analysis. KEGG analysis was performed using upregulated DEGs from baseline Inflammatory monocytes of all cohorts when compared to monocytes isolated from young healthy individuals. Dot plots represent significantly upregulated KEGG pathways with FDR of < 1% compared to young healthy individuals. The size of the node represents the enrichment factor defined by overlap of the input to the gene set; color of node represents -Log10(FDR).

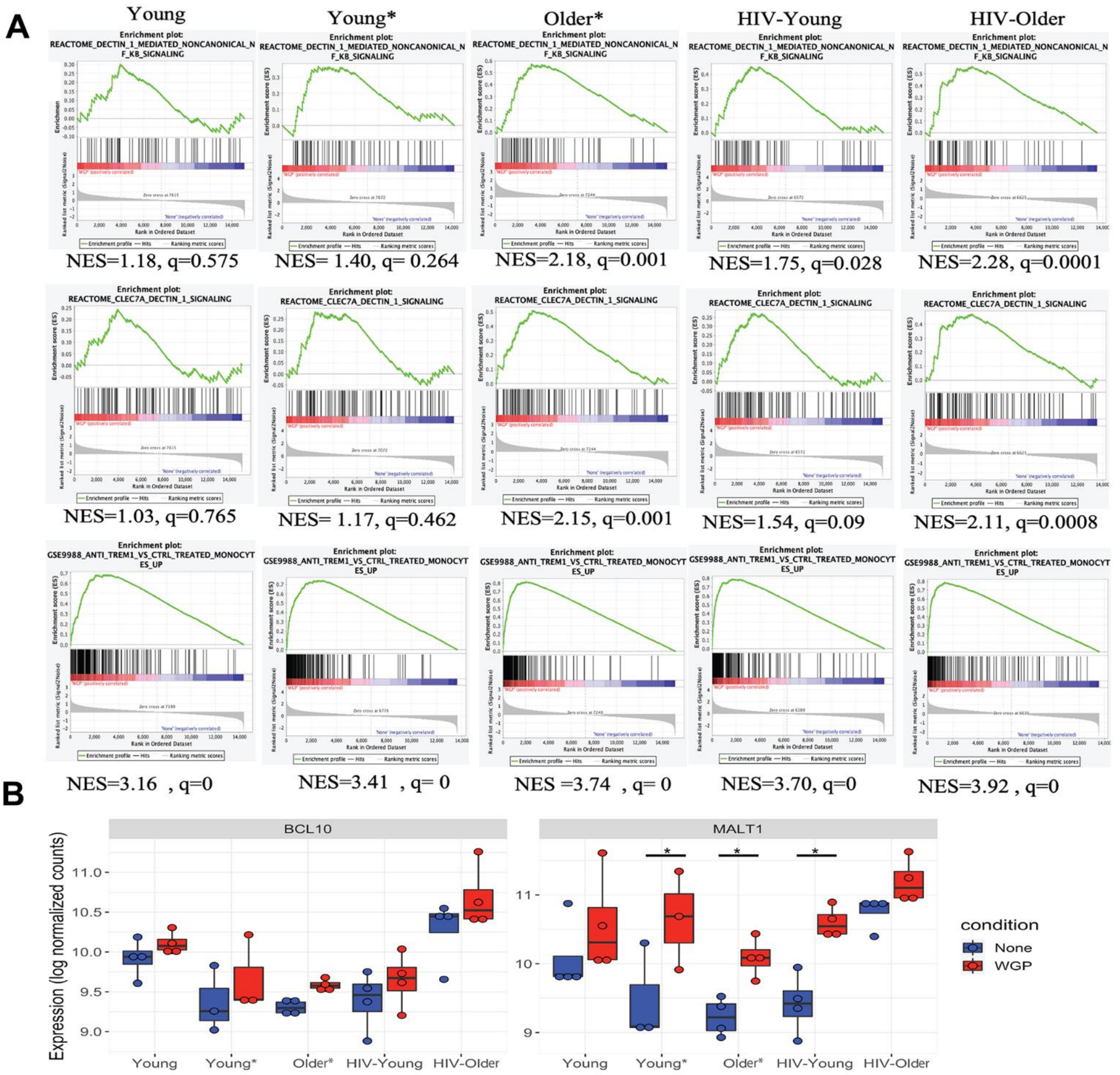

Supplementary Figure 9. Activation of Dectin-1/CLEC7A and TREM1 signaling in inflammatory monocytes. Gene set enrichment analysis (GSEA) was performed on normalized counts from all cohorts independently. Enrichment plots are shown. (**A**) Reactome Dectin-1 mediated noncanonical NF-kB signaling, Reactome CLEC7A Dectin-1 signaling and ImmuneSigDB TREM-1 signaling in monocytes. The respective normalized enrichment scores (NES) and q-values (FDR) are mentioned for each group. The scores towards the left side (red) represent the gene sets positively correlated with WGP, while scores towards the right side (blue) represent the gene sets negatively correlated with WGP. (**B**) Bcl10 and MALT1 are upregulated in all cohorts. Boxplots showing log normalized counts of BCL10 and MALT1 in the unstimulated and WGP stimulated CD14+CD16+ monocytes. The symbol * represents significant differentially expressed genes with fold cutoff of 1.2, and q value < 0.1.

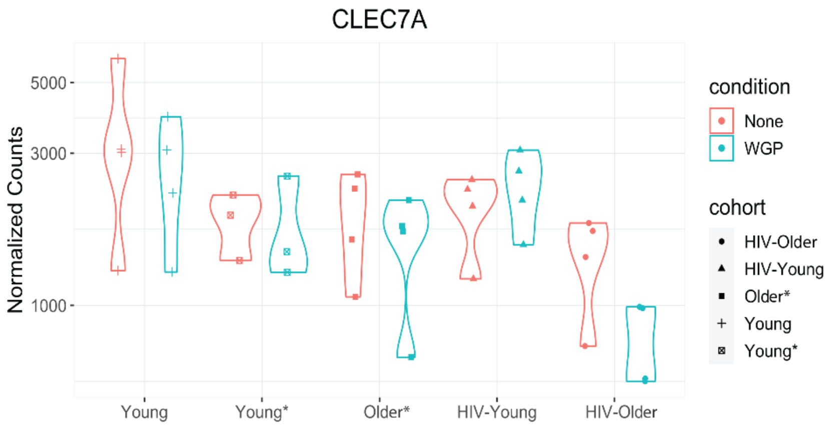

Supplementary Figure 10. CLEC7A (Dectin-1) expression. Violin plot is shown representing normalized counts of CLEC7A gene expression in unstimulated and WGP stimulated CD14+CD16+ monocytes among all cohorts. The plotCounts function of DEseq2 was used to plot counts. Each point represents an individual.

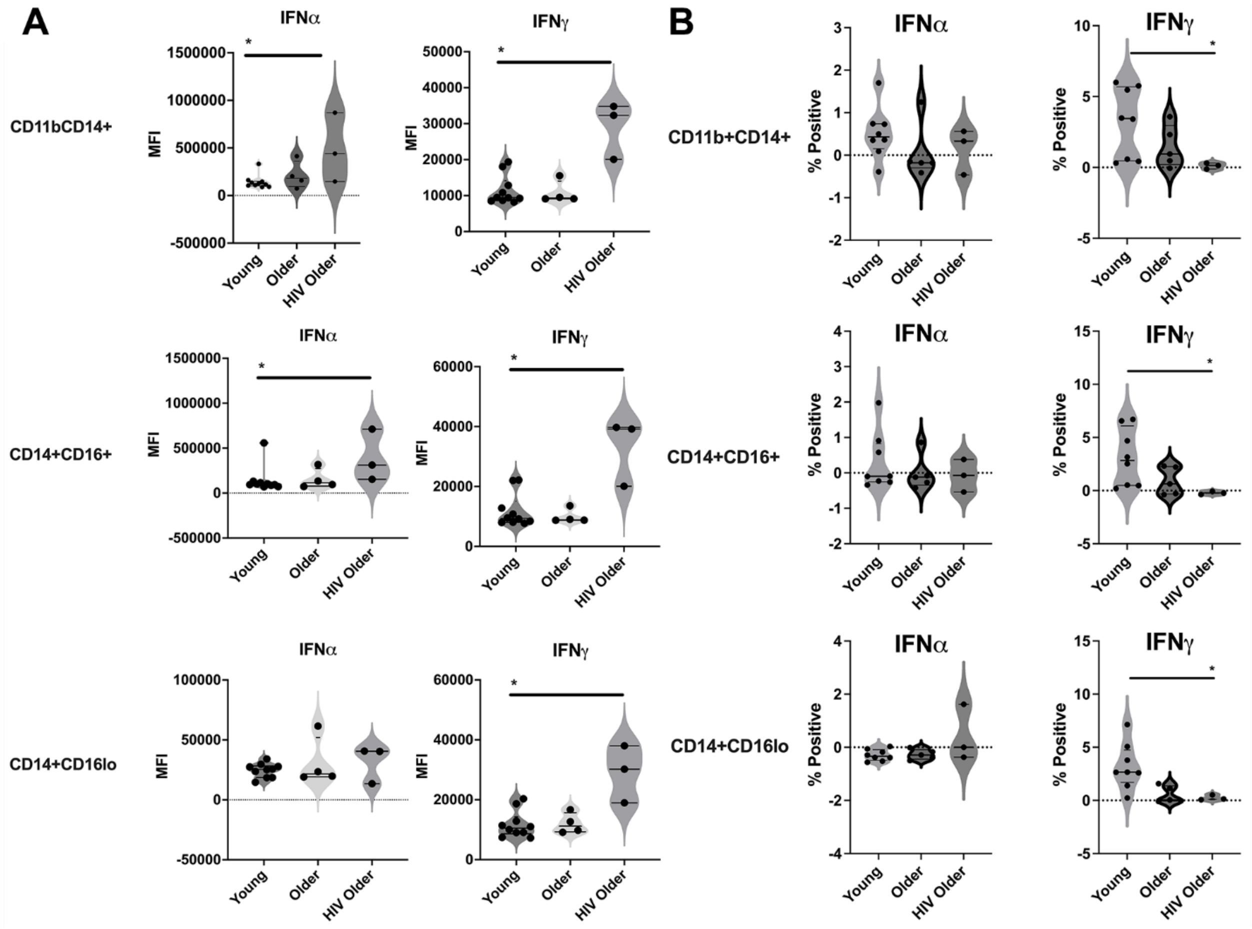

Supplementary Figure 11. IFN-α and IFN-γ production in monocytes. HIV-negative young adults (Young) (n=8), HIV-negative older adults (Older) (n= 4), HIV-positive older (HIV-Older) (n=3). Comparisons were analyzed using a Mann-Whitney test. All other comparisons were not found to be significant. (**A**) Baseline values: CD11b+CD14+: [IFN-α], Young vs. HIV-Older, (p= 0.028), [IFN-γ] HIV-Older vs. Young, (p=0.007). All other values were non-significant. [CD14+CD16+]: [IFN-α], Young vs. HIV-Older, (p= 0.024), [IFN- γ], Young vs. HIV-Older, (p= 0.028). CD14+CD16lo: [IFN-γ], Young vs. HIV-Older, (p= 0.014). (**B**) Dectin-1 stimulation: CD11b+CD14+, [IFN-γ], Young vs. HIV-Older, (p= 0.018). CD14+CD16+, [IFN-γ], Young vs. HIV-Older, (p= 0.012). CD14+CD16lo, [IFN-γ], Young vs. HIV-Older, (p= 0.024).

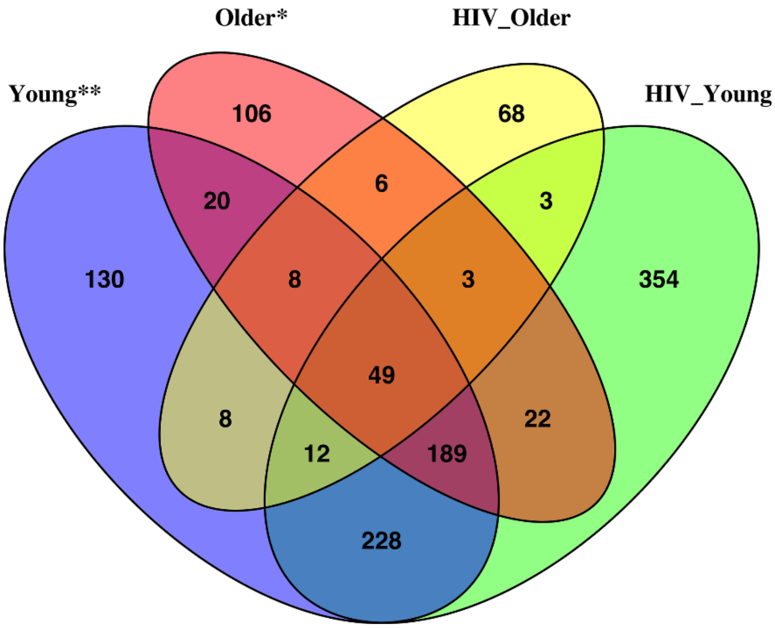

Supplementary Figure 12. Venn diagram of differentially expressed genes and shared genes amongst cohorts. A Venn diagram was created using DEGs (WGP vs unstimulated) from each cohort. For better visualization and understanding, the DEGs from the Young and Young* cohorts were merged and represented as Young** in periwinkle color. The DEGs from the Older*, HIV-Young, and HIV-Older cohorts are represented in pink, green, and yellow, respectively, and have exclusive counts of 106, 354, and 68 DEGs, respectively. Only 49 DEGs (burnt orange) were common across all cohorts after Dectin-1 stimulation. The DEGs common between respective two groups includes: 228 DEGs (blue) between Young** and HIV-Young, 22 DEGs (brown) between Older* and HIV-Young, 20 DEGs (dark pink) between Young** and Older*, 8 DEGs (olive) between Young** and HIV-Older, 6 DEGs (orange) between Older* and HIV-Older, 3 DEGs (light green) between HIV-Young and HIV-Older. The DEGs common among three groups include: 189 DEGs (purple) between Young** and Older* and HIV-Young, 12 DEGs (sage green) between Young** and HIV-Young and HIV-Older, 8 DEGs (melon) between Young** and Older* and HIV-Older, 3 DEGs (mustard) between Older* and HIV-Young and HIV-Older. An accompanying Supplementary Table 5 lists all gene categories.

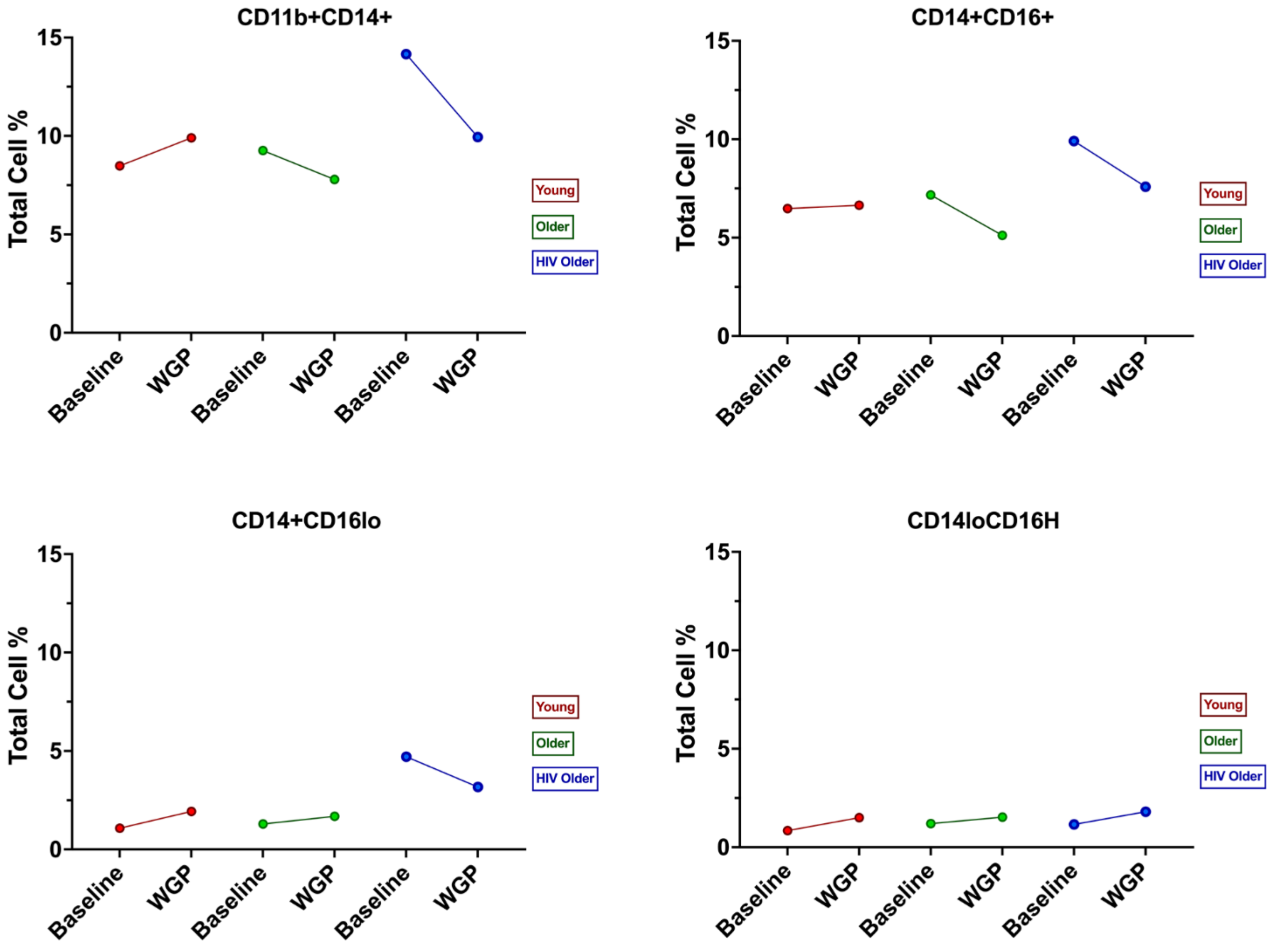

Supplementary Figure 13. Cell numbers before and after stimulation. Total cell percentages before and after WGP stimulation. Total monocyte subsets percentages (CD11b+CD14+, CD14+CD16+, CD14+CD16lo, CD14+CD16Hi) are plotted in comparison to WGP stimulated populations within each patient cohort; Young (red), Older (green) and HIV-Older (blue). Most numbers were comparable before and after stimulation. The only substantial differences were noted in the HIV Older cohort in the activated (4.2%) and inflammatory monocyte (2.3%) subsets where we saw less than 5% drop in cell number.

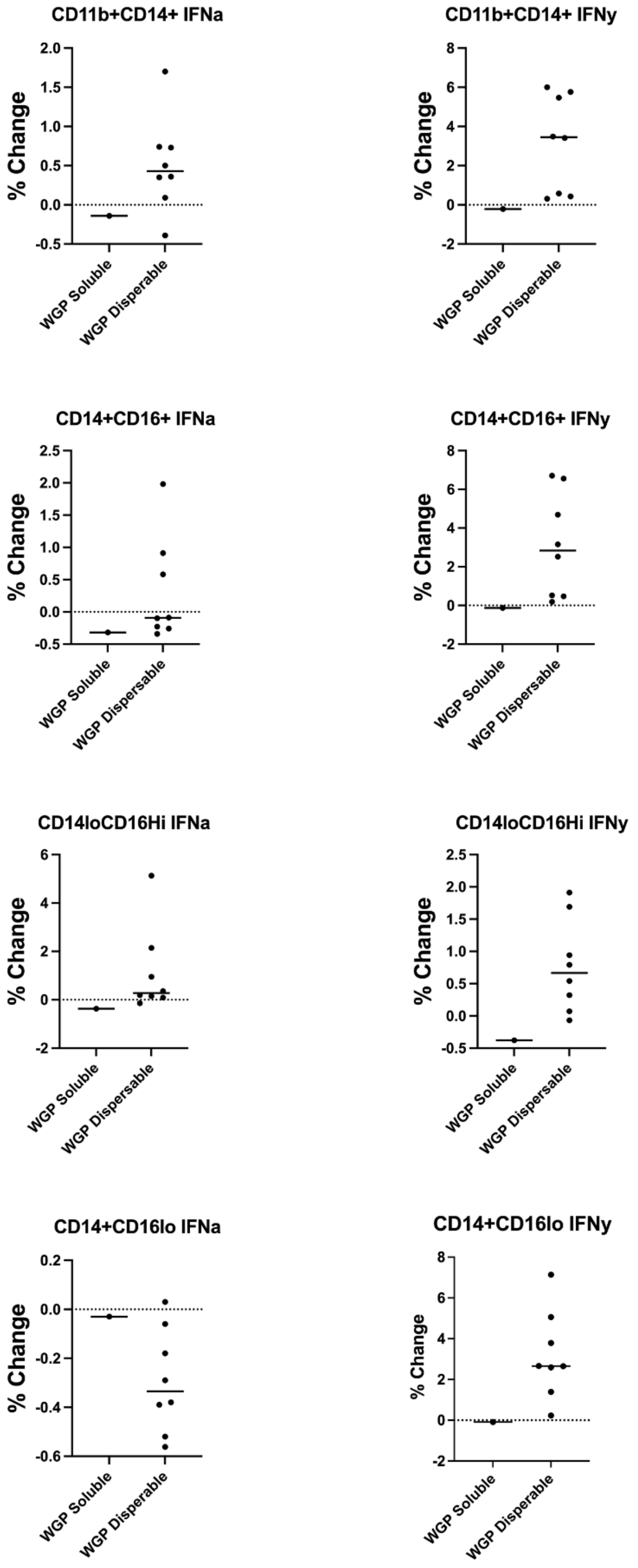

Supplementary Figure 14. WGP soluble control. Comparison of % change in cytokine production when WGP soluble (used as a negative control) is compared to the WGP dispersible ligand. Shown is a representative sample of our data.

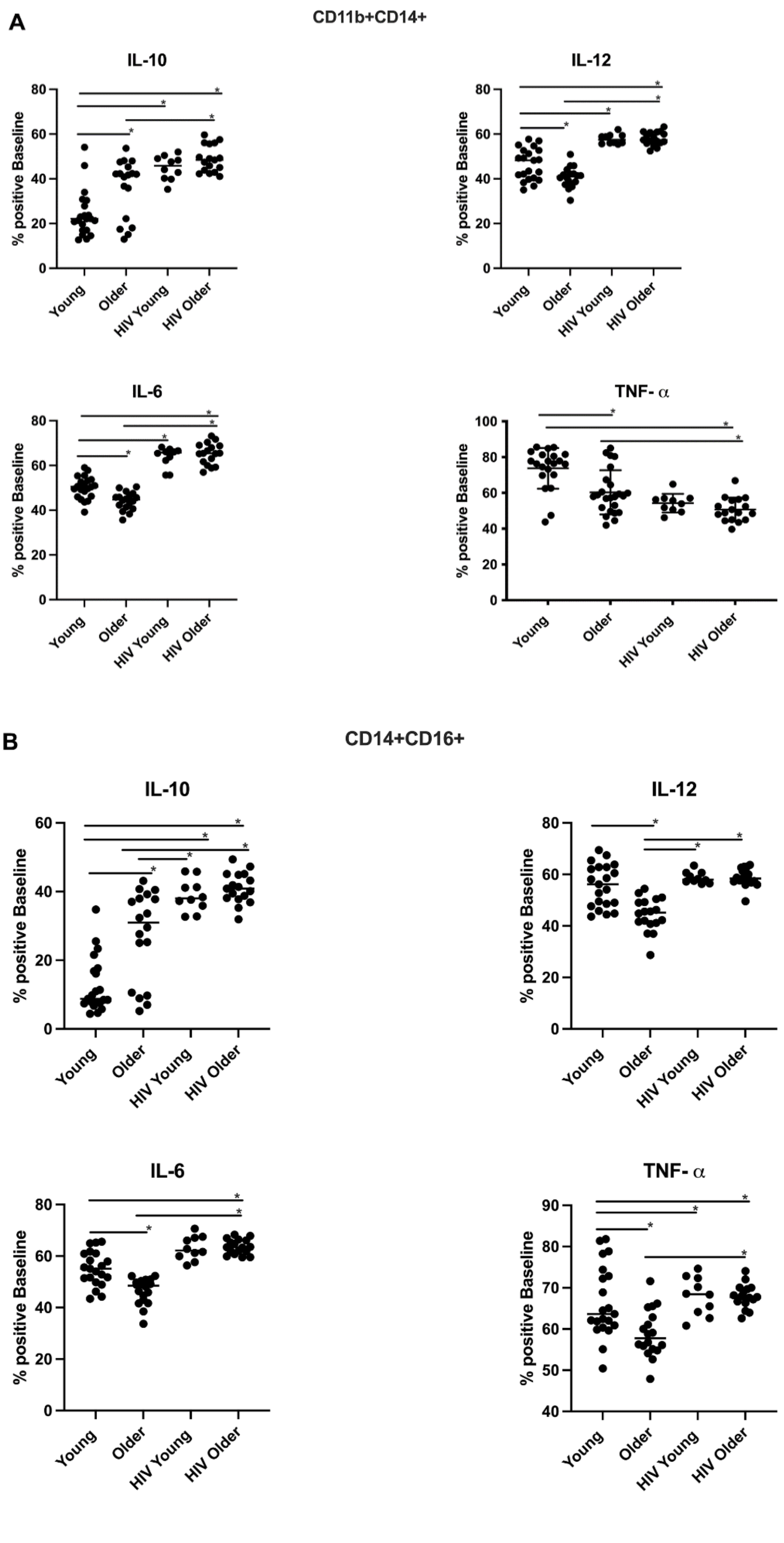

Supplementary Figure 15. Baseline (% positive) cytokine data for CD11b+CD14+ and CD14+CD16+ monocytes. (**A**) CD11b+CD14+ [IL-10]: Young vs. Older, (p=0.0105), Young vs. HIV-Young (p<0.0001), Young vs. HIV-Older (p<0.0001), Older vs. HIV-Older

(p=0.0004). [TNF-α]: Young vs. Older (p=0.0010), Young vs. HIV-Older (p<0.0001), Young vs. HIV-Young (p=0.0001), Older vs. HIV-Older (p=0.0030). [IL-12]: Young vs. Older, (p=0.0123), Young vs. HIV-Older (p<0.0001), Older vs. HIV-Older (p<0.0001). [IL-6]: Young vs. Older (p=0.0003), Young vs. HIV-Young (p<0.0001), Young vs. HIV-Older (p<0.0001), Older vs. HIV-Older (p<0.0001). (**B**) CD14+CD16+ [IL-10]: Young vs. Older (p=0.008), Young vs. HIV-Young (p<0.0001), Young vs. HIV-Older, (p<0.0001), Older vs. HIV-Older, (p=0.0002), Older vs. HIV-Younger, (p=0.0181). [TNF-α]: Young vs. Older, (p=0.0001), Young vs. HIV-Young, (p=0.0326), Young vs. HIV-Older, (p=0.03), Older vs. HIV-Older, (p<0.0001). [IL-12]: Young vs. Older, (p=0.0001), Older vs. HIV-Young, (p<0.0001), Older vs. HIV-Older (p<0.0001). [IL-6]: Young vs. Older, (p<0.0001), Young vs. HIV-Older, (p<0.0001), Older vs. HIV-Older, (p<0.0001). Comparisons were analyzed using a Mann-Whitney test. All other comparisons were not significant.

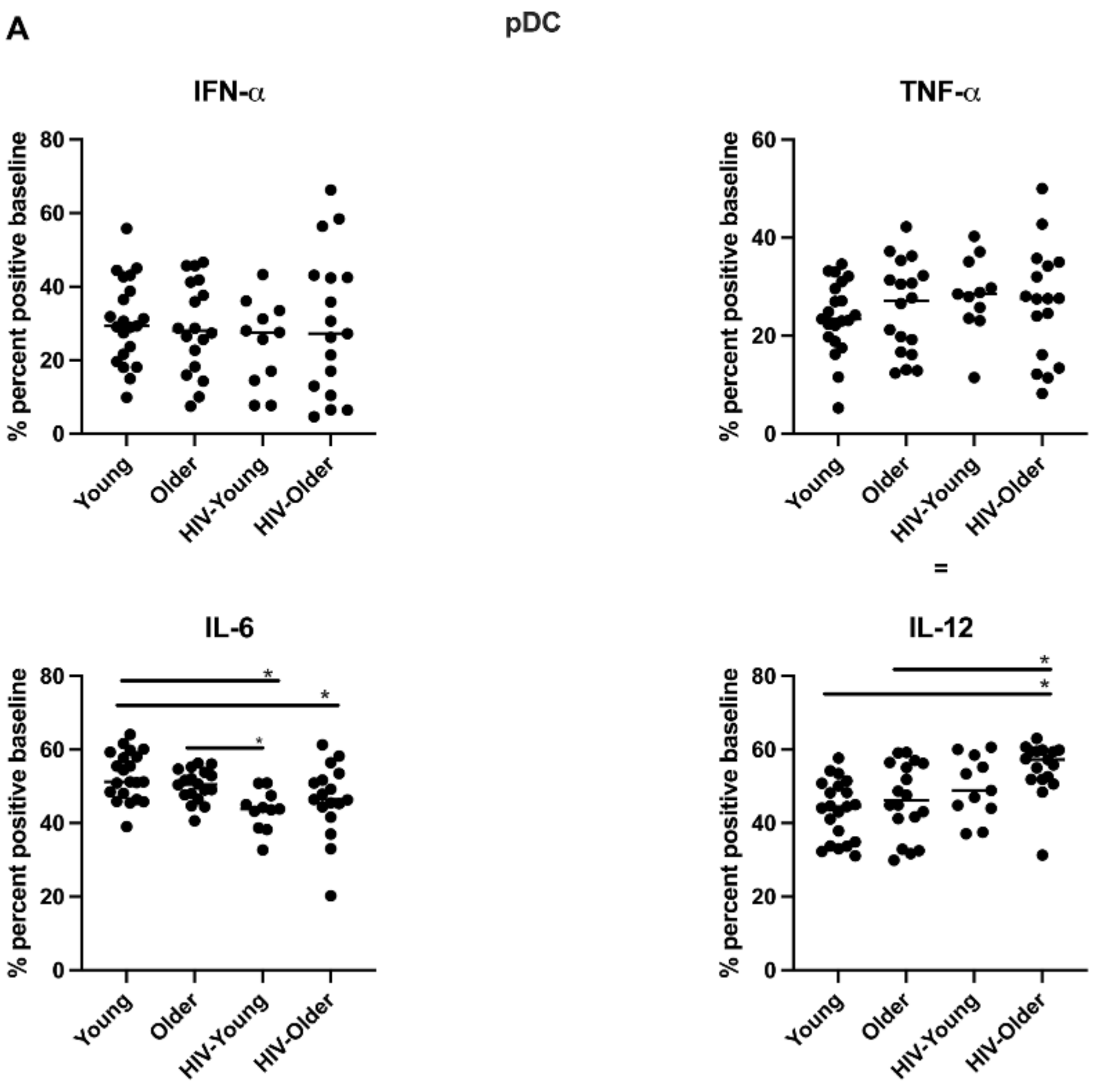

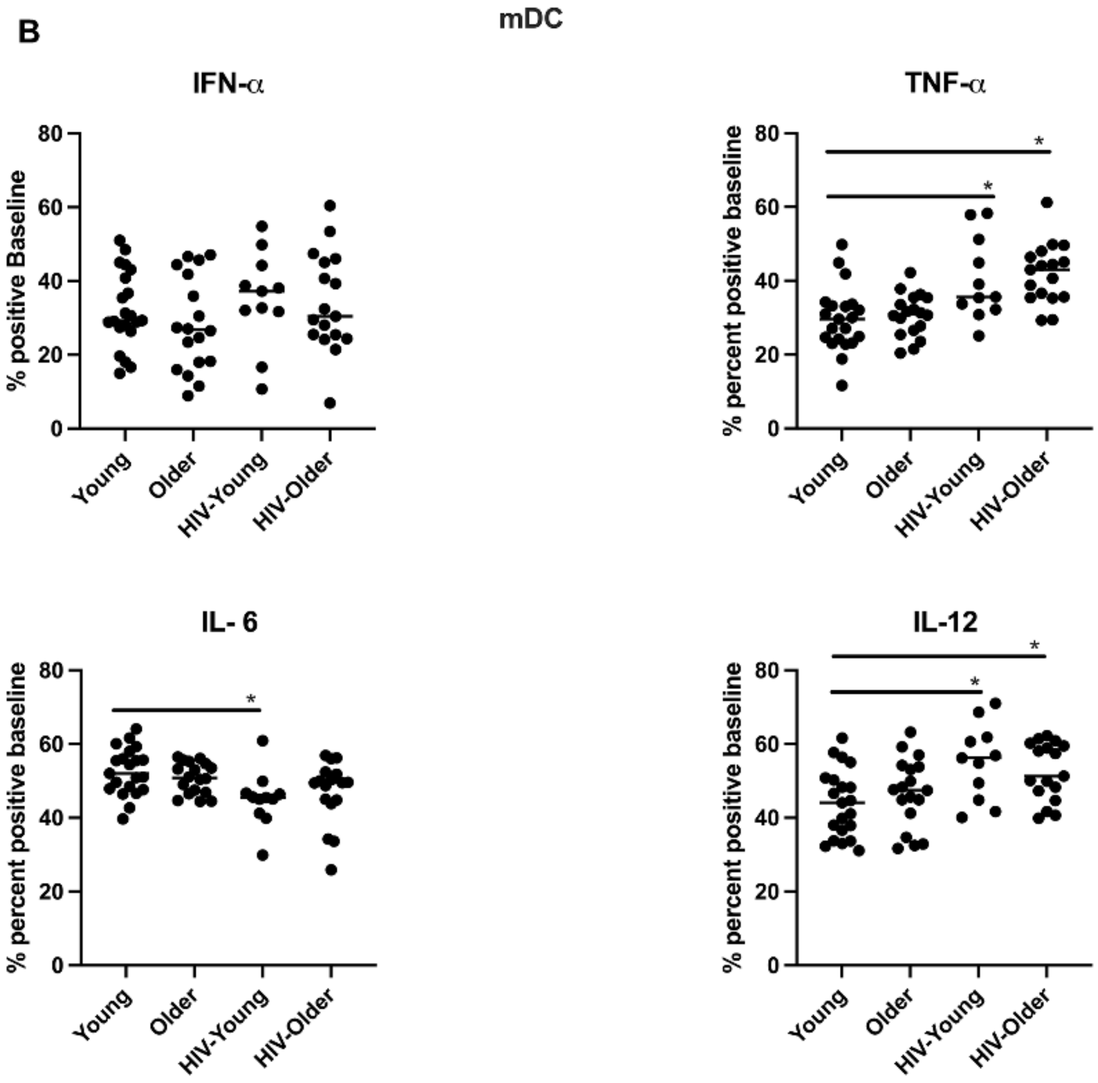

Supplementary Figure 16. Baseline (% positive) cytokine data for dendritic cells. (**A**) mDC [TNF-α]: Young vs. HIV-Young, (p=0.0033), Young vs. HIV-Older, (p<0.0001). [IL-6]: Young vs. HIV-Young, (p=0.0033). [IL-12]: Young vs. HIV-Young, (p=0.0064), Young vs. HIV-Older, (p=0.0045). (**B**) pDC [IL-6]: Young vs. HIV-Young, (p=0.0001), Young vs. HIV-Older, (p=0.0269), Older vs. HIV-Young, (p=0.0015). [IL-12]: Young vs. HIV-Older, (p<0.0001), Older vs HIV-Older, (p=0.0033). Comparisons were analyzed using a Mann-Whitney test. All other comparisons were not significant.
