# Supplementary material for: Dectin-1 stimulation promotes a distinct inflammatory signature in the setting of HIV-infection and aging
Source: Aging (Albany NY). 2023 Aug 21;15(16):7866–908. doi: 10.18632/aging.204927 (PMC10497004; doi:10.18632/aging.204927)
Supplement: Supplementary Table 1 [file aging-15-204927-s002.docx]

**Supplementary Table 1**. Upstream regulator prediction was made using QIAGEN Ingenuity Pathway Analysis (IPA). DEGs were analyzed for upstream regulators using IPA. The upstream regulators with Z-score of +2 are considered as significant. The upstream regulator and their Z-scores are listed for each cohorts.

| **Upstream Regulators** | **Young** | **PCC-Young** | **PCC-Old** | **HIV-Young** | **HIV-Old** |
| --- | --- | --- | --- | --- | --- |
| BCR (complex) | 3.1 | 2.219 | 3.257 | 2.952 | N/A |
| MAPK1 | 1.757 | 2.213 | 1.876 | 5.383 | N/A |
| IL15 | 2.442 | 2.778 | 2.414 | 3.256 | N/A |
| ASPSCR1-TFE3 | 2.236 | 3 | 2.449 | 3 | N/A |
| MMP1 | 2.4 | 2.598 | 2.598 | 2.954 | N/A |
| ADORA3 | 2.646 | 2.449 | 3 | 1.89 | N/A |
| KRAS | 2.449 | 2.449 | 2.236 | 2.772 | N/A |
| EZH2 | 3.293 | 1.34 | 2.834 | 2.424 | N/A |
| IL5 | 2.219 | 2.433 | 2.433 | 2.621 | N/A |
| let-7 | -2.066 | -2.643 | -2.25 | -2.59 | N/A |
| COL18A1 | -2.897 | -2.354 | -2.36 | -1.884 | N/A |
| PDCD4 | -2.621 | -1.982 | -2.425 | -2.425 | N/A |
| mir-8 | -2.236 | -1.987 | -2.438 | -2.407 | N/A |
| PAF1 | 2.433 | 2.236 | 2.63 | -1.698 | N/A |
| STAT5a/b | 2.219 | 2.425 | 2.213 | 1.937 | N/A |
| CRP | 2.191 | 1.995 | 2.191 | 2.412 | N/A |
| miR-146a-5p (and other miRNAs w/seed GAGAACU) | -1.664 | -2.433 | -2.219 | -2.433 | N/A |
| SP1 | 2.157 | 1.976 | 2.767 | 1.747 | N/A |
| PI3K (complex) | 2.371 | 1.746 | 2.573 | 1.926 | N/A |
| ERG | 1.914 | 2.339 | 2.186 | 2.037 | N/A |
| BRD4 | 2.345 | 2.561 | 2.557 | 0.98 | N/A |
| CBX5 | -2.121 | -2.646 | -2.236 | -1.387 | N/A |
| ATF4 | 1.98 | 1.478 | 2.391 | 2.432 | N/A |
| RAC1 | 2.236 | 1.89 | 1.89 | 2.121 | N/A |
| ETV1 | 2 | 2 | 2 | 2 | N/A |
| RNASE1 | 2.63 | 2.236 | 2.813 | -0.128 | N/A |
| RNASE2 | 2.449 | 2.204 | 2.779 | 0.286 | N/A |
| VEGFA | 2.383 | 1.679 | 1.764 | 1.871 | N/A |
| FOXM1 | 2.726 | 1.033 | 1.933 | 1.994 | N/A |
| ATM | 1.284 | 1.657 | 2.568 | 1.865 | N/A |
| SAA | 2.391 | 2.391 | N/A | 2.391 | N/A |
| Lymphotoxin | 2.449 | N/A | 2.449 | 2.236 | N/A |
| CEBPB | 2.165 | 1.183 | 2.619 | 1.065 | N/A |
| NR5A2 | 2.234 | 1.506 | 1.506 | 1.765 | N/A |
| PTAFR | 1.997 | 0.905 | 2.218 | 1.831 | N/A |
| TLR9 | 1.564 | 0.806 | 3.065 | -1.487 | N/A |
| CIC | N/A | -2.449 | -2.236 | -2.236 | N/A |
| SREBF1 | 1.934 | 1.346 | 2.166 | 1.46 | N/A |
| NEDD9 | 2 | 1.195 | 2.236 | 1.463 | N/A |
| FCER1G | 2.219 | N/A | 2.433 | 2.236 | N/A |
| PDPK1 | -2.198 | N/A | -2.408 | -2.225 | N/A |
| FCGR2A | 2.225 | N/A | 2.411 | 2.195 | N/A |
| ETS1 | 2.22 | 2.63 | N/A | 1.968 | N/A |
| NORAD | N/A | 2.236 | 2.219 | 2.345 | N/A |
| PIN1 | 2.191 | N/A | 2.201 | 2.408 | N/A |
| SIRT6 | 2.19 | N/A | 2.395 | 2.19 | N/A |
| IFNL1 | N/A | N/A | 1.94 | -4.821 | N/A |
| CREB1 | 2.42 | N/A | 2.041 | 2.224 | N/A |
| CD14 | 2.215 | N/A | 2.431 | 1.981 | N/A |
| TLR6 | 1.982 | N/A | 2.425 | 2.213 | N/A |
| Lh | 2.195 | 0.878 | 2.606 | 0.94 | N/A |
| TERT | N/A | 1.98 | 2.425 | 2.213 | N/A |
| Nr1h | -2.044 | N/A | -2.276 | -2.236 | N/A |
| IKBKB | 1.963 | N/A | 2.6 | 1.981 | N/A |
| IL1 | 1.982 | N/A | 2.219 | 2.219 | N/A |
| TRAF6 | 1.982 | N/A | 2.433 | 2 | N/A |
| PLAUR | 1.981 | 2.207 | N/A | 2.207 | N/A |
| MAPK8 | 2.2 | 1.423 | 1.095 | 1.664 | N/A |
| PDLIM2 | 2.121 | 1.134 | 1.897 | 1.213 | N/A |
| MAPK14 | 2.2 | N/A | 2.2 | 1.964 | N/A |
| CHUK | 2.213 | N/A | 2.4 | 1.746 | N/A |
| TLR5 | 1.966 | N/A | 2.407 | 1.98 | N/A |
| TLR1 | 1.969 | N/A | 2.401 | 1.972 | N/A |
| EHF | 2.449 | 1.265 | 1.89 | 0.707 | N/A |
| FIRRE | 2 | N/A | 2 | 2.236 | N/A |
| FASN | -1.964 | N/A | -2.236 | -2 | N/A |
| PCDH11Y | 1.982 | 1.982 | N/A | 2.213 | N/A |
| FAT1 | 2.213 | N/A | 1.974 | 1.974 | N/A |
| THRB | -1.455 | -1.501 | -2 | -1.2 | N/A |
| KDM3A | N/A | 2.19 | 1.96 | 1.969 | N/A |
| APP | 2.4 | N/A | 2.4 | 1.303 | N/A |
| EDN1 | 1.954 | N/A | 2.183 | 1.954 | N/A |
| MACROH2A1 | -2.121 | -0.816 | -2.333 | -0.707 | N/A |
| CRNDE | 2.236 | 1.969 | 0.509 | 1.242 | N/A |
| EHMT1 | 2.236 | 2.111 | 0.447 | 1.155 | N/A |
| mir-146 | -1.96 | N/A | -2.407 | -1.452 | N/A |
| MUC1 | 1.785 | 2.176 | 0.584 | 1.22 | N/A |
| FSH | 1.989 | 0.626 | 2.434 | 0.698 | N/A |
| IL33 | 2.213 | 0.689 | 1.392 | 1.443 | N/A |
| ABL1 | N/A | 1.951 | 2.186 | 1.406 | N/A |
| TFRC | -2.4 | -1.23 | N/A | -1.723 | N/A |
| P2RX7 | 2.2 | N/A | 1.96 | 1.131 | N/A |
| EFNA1 | -1 | -1.89 | N/A | -2.333 | N/A |
| RBPJ | N/A | -2.646 | N/A | -2.449 | N/A |
| EFNA2 | -1 | -1.89 | N/A | -2.121 | N/A |
| MAFB | N/A | -1.89 | N/A | -3.051 | N/A |
| SRC | 1.982 | 0.13 | 2.156 | 0.656 | N/A |
| WNT5A | 1.308 | N/A | 2.211 | 1.34 | N/A |
| ESRRA | 2.421 | N/A | N/A | 2.421 | N/A |
| E2F1 | N/A | N/A | 2.415 | 2.415 | N/A |
| IRF4 | -1.492 | -2 | -0.213 | 0.784 | N/A |
| PRKAA1 | 1.067 | N/A | 1.387 | 2 | N/A |
| PRKCE | 2.164 | N/A | 0.964 | 1.304 | N/A |
| JAK1 | 2 | N/A | 2 | 0.378 | N/A |
| IRGM | N/A | N/A | N/A | 4.345 | N/A |
| SOX11 | N/A | 1.912 | N/A | 2.331 | N/A |
| PGF | N/A | N/A | 2.229 | 1.994 | N/A |
| IFIH1 | 1.987 | N/A | 2.216 | N/A | N/A |
| NR1H4 | -1.964 | N/A | -2.2 | N/A | N/A |
| ELAVL1 | N/A | N/A | 2.401 | 1.747 | N/A |
| BCL2L1 | -2.219 | -1 | N/A | -0.896 | N/A |
| SCD | N/A | N/A | -2.157 | -1.934 | N/A |
| RNY3 | N/A | N/A | N/A | -4.025 | N/A |
| HDAC1 | N/A | -2 | N/A | -2 | N/A |
| miR-182-5p (and other miRNAs w/seed UUGGCAA) | N/A | N/A | N/A | 3.86 | N/A |
| KMT2D | N/A | 2.224 | N/A | 1.59 | N/A |
| CBFA2T3 | 0.659 | 0.659 | N/A | 2.484 | N/A |
| miR-450a-5p (and other miRNAs w/seed UUUGCGA) | N/A | -2.236 | N/A | -1.414 | N/A |
| EZR | N/A | N/A | -2 | -1.633 | N/A |
| TLR8 | N/A | N/A | 2.422 | 1.177 | N/A |
| IL1RAP | N/A | N/A | 1.342 | 2 | N/A |
| RC3H1 | N/A | N/A | N/A | 3.207 | N/A |
| IFNB1 | N/A | N/A | N/A | -3.144 | N/A |
| CGAS | N/A | N/A | 2.213 | 0.796 | N/A |
| IFNL4 | N/A | N/A | N/A | -2.975 | N/A |
| IFNAR2 | N/A | N/A | N/A | -2.646 | N/A |
| JAK | N/A | N/A | N/A | -2.646 | N/A |
| SOCS1 | 0.192 | N/A | 0.192 | 2.177 | N/A |
| miR-122-5p (miRNAs w/seed GGAGUGU) | N/A | -2.449 | N/A | N/A | N/A |
| TSLP | N/A | N/A | 2.425 | N/A | N/A |
| IRF1 | N/A | N/A | N/A | -2.39 | N/A |
| miR-511-5p (miRNAs w/seed UGUCUUU) | N/A | N/A | -2.236 | N/A | N/A |
| ANLN | N/A | N/A | N/A | -2.236 | N/A |
| AURK | N/A | N/A | N/A | -2.219 | N/A |
| HDAC4 | N/A | N/A | N/A | -2.219 | N/A |
| miR-296-5p (miRNAs w/seed GGGCCCC) | N/A | -2.219 | N/A | N/A | N/A |
| GATA6 | N/A | N/A | N/A | 2.213 | N/A |
| IRF7 | N/A | N/A | N/A | -2.202 | N/A |
| ISG15 | N/A | N/A | N/A | 2.195 | N/A |
| IKZF3 | N/A | N/A | N/A | 2.137 | N/A |
| TAP1 | N/A | N/A | -2 | 0.112 | N/A |
| HMGB1 | N/A | N/A | 2.085 | N/A | N/A |
| PAEP | N/A | N/A | -2 | N/A | N/A |
| RUNX1 | 2 | N/A | N/A | N/A | N/A |
| WNT7A | N/A | N/A | N/A | 2 | N/A |
| NFIX | N/A | N/A | N/A | -2 | N/A |
| FCER2 | N/A | N/A | 2 | N/A | N/A |
| FENDRR | 2 | N/A | N/A | N/A | N/A |
| CLEC4E | N/A | N/A | 2 | N/A | N/A |
| LGALS8 | N/A | N/A | 2 | N/A | N/A |
| TPR | N/A | N/A | 2 | N/A | N/A |
| MAP2K3 | N/A | N/A | N/A | -2 | N/A |
| TREM1 | 7.147 | 6.929 | 7.484 | 7.617 | 5.785 |
| TNF | 5.777 | 4.211 | 6.326 | 3.016 | 4.193 |
| SMARCA4 | 5.043 | 4.483 | 4.745 | 3.872 | 4.123 |
| IL1B | 4.699 | 3.941 | 5.19 | 3.52 | 3.639 |
| RELA | 3.73 | 1.992 | 4.507 | 1.252 | 3.582 |
| FOXO1 | 2.926 | 2.596 | 2.486 | 1.86 | 3.257 |
| IL1A | 4.227 | 2.725 | 4.423 | 3.152 | 3.2 |
| IL17A | 3.89 | 2.395 | 3.872 | 2.847 | 3.197 |
| NFkB (complex) | 4.457 | 2.855 | 5.05 | 2.086 | 3.196 |
| PDGF BB | 4.783 | 4.125 | 4.911 | 4.794 | 3.162 |
| F7 | 3.13 | 2.449 | 3.108 | 2.931 | 2.945 |
| PRKCD | 3.199 | 2.944 | 3.483 | 3.281 | 2.92 |
| IFNG | 2.189 | 0.321 | 3.235 | -3.362 | 2.905 |
| JUN | 3.53 | 3.09 | 4.175 | 4.027 | 2.786 |
| OSCAR | 3.742 | 3.317 | 3.742 | 3.606 | 2.646 |
| CCL5 | 2.828 | 3.317 | 3.606 | 2.84 | 2.646 |
| SYVN1 | 3 | 2.53 | 3.317 | 2 | 2.646 |
| CHD1 | 3.464 | N/A | 3.317 | 2.646 | 2.646 |
| TGFB1 | 3.653 | 3.519 | 3.652 | 3.084 | 2.618 |
| CD28 | 2.965 | 1.916 | 2.589 | 2.402 | 2.594 |
| P38 MAPK | 3.178 | 2.576 | 4.007 | 1.276 | 2.586 |
| TLR7 | 2.15 | 0.265 | 3.775 | -1.094 | 2.57 |
| EGF | 2.492 | 2.819 | 3.522 | 3.303 | 2.564 |
| FOXO3 | 1.377 | 0.911 | 2.169 | 0.161 | 2.538 |
| GPER1 | 3.286 | 3.13 | 3.973 | 3.576 | 2.449 |
| Collagen type II | 3.606 | 3.162 | 3.317 | 3.317 | 2.449 |
| RNF138 | 2.646 | N/A | 2.828 | 2 | 2.449 |
| CSF2 | 4.262 | 3.885 | 4.157 | 4.123 | 2.433 |
| FOXL2 | 3.293 | 2.635 | 2.885 | 3.145 | 2.429 |
| CD36 | 3.845 | 3.162 | 3.576 | 3.01 | 2.414 |
| Immunoglobulin | 2.534 | 1.778 | 3.434 | 0.671 | 2.414 |
| FOS | 1.718 | 1.414 | 2.714 | 2.496 | 2.414 |
| CD40LG | 2.61 | 1.007 | 3.794 | 2.007 | 2.406 |
| S100A8 | 2.572 | N/A | 2.921 | 1.985 | 2.388 |
| ERK1/2 | 3.198 | 0.996 | 3.507 | 2.197 | 2.387 |
| MAP2K1/2 | 2.739 | 2.92 | 3.36 | 3.361 | 2.38 |
| HIF1A | 2.538 | 0.331 | 3.451 | 1.215 | 2.356 |
| TGM2 | 3.576 | 2.323 | 2.891 | -0.561 | 2.345 |
| TLR4 | 2.869 | 2.547 | 3.166 | 1.975 | 2.339 |
| NUPR1 | 3.273 | 3.656 | 4.271 | 4.061 | 2.333 |
| STAT3 | 3.02 | 1.265 | 2.784 | 2.023 | 2.314 |
| TLR7/8 | 2.646 | 2.449 | 3.317 | 1.897 | 2.236 |
| MAP2K1 | 2.159 | 2.139 | 2.271 | 2.35 | 2.236 |
| SELPLG | 2.449 | 1.342 | 3 | 1.414 | 2.236 |
| MTOR | 1.913 | N/A | 1.238 | 0.798 | 2.236 |
| CD3 | 2.676 | 1.706 | 2.751 | 0.444 | 2.224 |
| SMAD4 | 1.551 | 1.303 | 1.57 | 1.805 | 2.224 |
| Interferon alpha | 1.712 | 1.731 | 0.518 | -3.088 | 2.219 |
| CAMP | 3.554 | 3.252 | 3.157 | 3.667 | 2.21 |
| PPRC1 | 3.111 | 2 | 2.771 | 3.266 | 2.197 |
| NCR2 | 2.779 | 2.186 | 2.95 | 2.4 | 2.19 |
| F2 | 2.215 | 1.706 | 2.752 | 1.522 | 2.177 |
| IL18 | 2.928 | 1.999 | 3.244 | 1.329 | 2.176 |
| EGFR | 2.986 | 3.111 | 3.005 | 3.279 | 2.17 |
| TLR2 | 3.212 | 3.061 | 3.338 | 3.091 | 2.156 |
| NFKB1 | 1.982 | 1.342 | 2.887 | 0.842 | 2.129 |
| CG | 1.978 | 1.348 | 3.104 | 0.612 | 2.023 |
| JUNB | 2.121 | 2.121 | 3 | 2.714 | 2 |
| TNFSF14 | 2.643 | 2.234 | 2.612 | 1.349 | 1.999 |
| PTGS2 | 2.229 | 1.778 | 2.42 | 1.603 | 1.992 |
| PLG | 2.423 | N/A | 2.614 | 2.207 | 1.991 |
| C5 | 2.109 | 1.857 | 2.438 | 2.332 | 1.987 |
| Cdk | 2.224 | N/A | 1.854 | 2 | 1.987 |
| NAMPT | 1.991 | 1.274 | 2.071 | 1.612 | 1.986 |
| CD3 group | 1.953 | 2.19 | 2.2 | 2.2 | 1.982 |
| CTNNB1 | 0.935 | 1.133 | 0.369 | 2.416 | 1.982 |
| Ap1 | 2.573 | 2.804 | 2.755 | 3.132 | 1.98 |
| ERK | 4.308 | 3.528 | 3.544 | 3.815 | 1.979 |
| PF4 | 2.309 | 0.522 | 2.992 | 1.634 | 1.963 |
| S100A9 | 2.374 | 1.934 | 2.576 | 2.377 | 1.962 |
| NFAT5 | 1.961 | 1.948 | 2.395 | 1.948 | 1.961 |
| TLR3 | 2.199 | N/A | 2.406 | -0.085 | 1.955 |
| GLI1 | 2.401 | 2.719 | 1.82 | 2.37 | 1.951 |
| MYD88 | N/A | N/A | 2.189 | 1.56 | 1.951 |
| EGR1 | 2.585 | 3.082 | 2.926 | 3.641 | 1.941 |
| FGF2 | 2.449 | 1.808 | 2.449 | 1.947 | 1.941 |
| ECSIT | 2.772 | N/A | 3.264 | 2.588 | 1.941 |
| BSG | 2.412 | 1.939 | 2.607 | 1.807 | 1.934 |
| Tlr | 1.93 | N/A | 2.152 | 1.929 | 1.929 |
| Fcer1 | 2.934 | 2.401 | 3.253 | 2.568 | 1.917 |
| PRL | 1.957 | N/A | 1.489 | -4.321 | 1.914 |
| PPARD | 3.317 | 2.236 | 2.704 | 2.646 | 1.913 |
| Akt | 1.995 | 1.227 | 2.497 | 1.631 | 1.896 |
| Mek | 2.683 | 2.112 | 3.113 | 1.504 | 1.856 |
| SPI1 | 0.555 | 1.206 | 1.151 | -3.358 | 1.732 |
| Jnk | 3.051 | 1.859 | 4.16 | 2.777 | 1.706 |
| TCR | 2.187 | 2.591 | 1.038 | 2.939 | 1.698 |
| IL2 | 1.593 | 2.157 | 1.62 | 2.739 | 1.514 |
| TP63 | 2.586 | 1.423 | 2.322 | 1.475 | 1.488 |
| ERBB2 | 3.058 | 3.482 | 3.234 | 2.87 | 1.405 |
| IFNA2 | N/A | N/A | N/A | -4.266 | 1.402 |
| IGF1 | 1.006 | 1.643 | 2.553 | 1.902 | 1.308 |
| IKBKE | 2.224 | 2.229 | 1.992 | 2.49 | 1.131 |
| CD40 | 1.948 | 2.256 | 2.825 | 2.362 | 1.066 |
| DDX58 | 2.414 | N/A | 2.056 | 1.44 | 0.958 |
| BTK | 1.47 | 1.616 | 0.488 | 3.983 | 0.818 |
| HGF | 2.416 | 0.286 | 2.313 | 1.352 | 0.804 |
| PGR | 1.167 | 1.906 | 1.074 | 3.741 | 0.726 |
| STAT1 | N/A | N/A | 1.737 | -3.711 | 0.718 |
| IL27 | 1.747 | 2.136 | 1.9 | -0.421 | 0.552 |
| IL4 | 2.738 | 3.326 | 3.315 | 3.573 | 0.468 |
| estrogen receptor | -2.284 | -1.888 | -1.119 | -2.049 | 0.447 |
| ESR1 | 1.429 | 1.337 | -0.056 | 2.272 | -0.404 |
| MYC | 0.897 | -0.422 | -0.471 | 2.505 | -0.529 |
| IL13 | 1.527 | 3.353 | 1.141 | 3.299 | -0.822 |
| IL10 | -0.932 | -1.253 | -1.745 | -2.316 | -0.937 |
| NEUROG1 | -1 | -1.508 | -2.449 | -0.832 | -1 |
| GSTO1 | -1.3 | -2.086 | -0.539 | -0.184 | -1.069 |
| Hdac | -1.873 | -2.097 | -2.524 | -2.023 | -1.564 |
| NR3C1 | -0.75 | -1.4 | -2.597 | -1.509 | -1.782 |
| IL37 | -2.18 | N/A | -2.38 | -2.378 | -1.944 |
| HLX | -0.849 | N/A | -2.213 | -0.762 | -1.982 |
| JAG2 | -2.915 | -2.433 | -2.395 | -1.964 | -2 |
| OGA | 0 | -1.36 | -2.359 | -1.422 | -2 |
| TAB1 | -1.982 | N/A | -1.387 | 0.707 | -2 |
| WBP2 | -1 | N/A | -1.897 | -0.277 | -2 |
| COPA | N/A | N/A | -2 | -1 | -2 |
| S100A6 | N/A | N/A | -1.89 | -1 | -2 |
| PDCD1 | N/A | -0.447 | -1.342 | -0.707 | -2 |
| ARID1A | N/A | N/A | -1.166 | -0.868 | -2 |
| CIP2A | -1.387 | -0.478 | -2.433 | -1.066 | -2.219 |
| miR-155-5p (miRNAs w/seed UAAUGCU) | -3.539 | -2.789 | -3.4 | -3.095 | -2.385 |
| IL1RN | -2.177 | -0.651 | -2.371 | 2.155 | -2.449 |
| ETV6-RUNX1 | -3 | -2.692 | -3.308 | -2.031 | -2.828 |
