# Supplementary material for: Dectin-1 stimulation promotes a distinct inflammatory signature in the setting of HIV-infection and aging
Source: Aging (Albany NY). 2023 Aug 21;15(16):7866–908. doi: 10.18632/aging.204927 (PMC10497004; doi:10.18632/aging.204927)
Supplement: Supplementary Tables 2-4 [file aging-15-204927-s003.pdf]

Supplementary Table 2. Clinical characteristics of RNA-seq participants.

	HIV-negative		HIV-positive		p
	Young	Older	Young	Older	
Female	4 (57.14)	2 (50.00)	3 (75.00)	0 (0.00)	0.256 [1]
Race					
Black	3 (42.86)	2 (50.00)	2 (50.00)	2 (50.00)	0.593 [1]
White	3 (42.86)	2 (50.00)	0 (0.00)	2 (50.00)	
Other	1 (14.29)	0 (0.00)	2 (50.00)	0 (0.00)	
Latinx	2 (28.57)	1 (25.00)	2 (50.00)	1 (25.00)	0.917 [1]
BMI Mean (SD)	27.1 (2.9)	35.0 (5.6)	29.2 (14.5)	26.7 (4.9)	0.624 [2]
BMI >= 30kg/m2	1 (14.29)	3 (75.00)	1 (25.00)	1 (25.00)	0.252 [1]
DM2	1 (14.29)	3 (75.00)	1 (25.00)	1 (25.00)	0.252 [1]
Metabolic Syndrome	0 (0.00)	4 (100.00)	1 (25.00)	0 (0.00)	0.002 [1]
CAD and/or PVD	0 (0.00)	0 (0.00)	0 (0.00)	2 (50.00)	0.105 [1]
Asthma and/or COPD	2 (28.57)	2 (50.00)	1 (25.00)	3 (75.00)	0.518 [1]
Autoimmune and/or autoinflammatory disease	1 (14.29)	0 (0.00)	0 (0.00)	0 (0.00)	1.000 [1]
Prior Smoker	1 (14.29)	0 (0.00)	0 (0.00)	0 (0.00)	1.000 [1]
Current Smoker	3 (42.86)	1 (25.00)	0 (0.00)	2 (50.00)	0.583 [1]
Recreational drug use in past year	3 (42.86)	0 (0.00)	1 (25.00)	1 (25.00)	0.711 [1]
History of positive PPD or Quantiferon	0 (0.00)	0 (0.00)	0 (0.00)	2 (50.00)	0.105 [1]
History of fungal disease	1 (14.29)	0 (0.00)	3 (75.00)	0 (0.00)	0.047 [1]
CD4 >200			4 (100.00)	4 (100.00)	NA [1]
VL > 100			1 (25.00)	0 (0.00)	1.000 [1]
on HAART			4 (100.00)	4 (100.00)	NA [1]
% life span with HIV [Mean (SD)]			63.3 (43.6)	44.0 (13.6)	0.008 [3]
Congenital HIV			2 (50.00)	0 (0.00)	0.429 [1]

[1]Fisher's Exact Test.
[2]ANOVA.
[3]T-test.
Fungal disease was defined as fungal infections other than superficial cutaneous infections (e.g. tinea pedis).
Abbreviations: BMI, body mass index; CAD, coronary artery disease; COPD, chronic obstructive pulmonary disease; DM, diabetes mellitus; HAART, highly active antiretroviral therapy; HIV, human immunodeficiency virus; HLD, hyperlipidemia; HTN, hypertension; PPD, purified protein derivative; PVD, peripheral vascular disease; VL, viral load.

Supplementary Table 3. Demographics of all cohorts stratified by both age and HIV-status.

		Monocytes					Dendritic cells				
		Younger		Older			Younger		Older		
		Negative	Positive	Negative	Positive	p-value	Negative	Positive	Negative	Positive	p-value
Gender	Male	8 (38.10)	8 (57.14)	12 (50.00)	13 (59.09)	0.534[1]	8 (38.10)	6 (60.00)	11 (61.11)	10 (58.82)	0.449[2]
	Female	13 (61.90)	6 (42.86)	12 (50.00)	9 (40.91)		13 (61.90)	4 (40.00)	7 (38.89)	7 (41.18)	
Race/Ethnicity	Hispanic	1 (4.76)	5 (35.71)	2 (8.33)	5 (22.73)	0.057[2]	1 (4.76)	4 (40.00)	0 (0.00)	1 (5.88)	0.004[2]
	White	16 (76.19)	4 (28.57)	17 (70.83)	10 (45.45)	0.000[2]	16 (76.19)	2 (20.00)	16 (88.89)	11 (64.71)	0.000[2]
	Black	1 (4.76)	6 (42.86)	5 (20.83)	9 (40.91)	0.000[2]	1 (4.76)	4 (40.00)	2 (11.11)	5 (29.41)	
	Asian	4 (19.05)	0 (0.00)	0 (0.00)	0 (0.00)	0.000[2]	4 (19.05)	0 (0.00)	0 (0.00)	0 (0.00)	
	Other	0 (0.00)	4 (28.57)	2 (8.33)	3 (13.64)	0.000[2]	0 (0.00)	4 (40.00)	0 (0.00)	1 (5.88)	
Smoking		1 (4.76)	5 (35.71)	4 (16.67)	4 (18.18)	0.126[2]	1 (4.76)	3 (30.00)	2 (11.11)	4 (23.53)	0.183[2]
Recreational Drugs		1 (4.76)	5 (35.71)	1 (4.17)	6 (27.27)	0.011[2]	1 (4.76)	3 (30.00)	0 (0.00)	6 (35.29)	0.004[2]
Fungal Disease		0 (0.00)	1 (7.14)	0 (0.00)	3 (13.64)	0.081[2]	0 (0.00)	1 (10.00)	0 (0.00)	6 (35.29)	0.001[2]
Number of Comorbidities	0	5 (23.81)	0 (0.00)	1 (4.17)	0 (0.00)	0.000[2]	5 (23.81)	0 (0.00)	1 (5.56)	0 (0.00)	0.000[2]
	1-3	15 (71.43)	7 (50.00)	2 (8.33)	3 (13.64)		15 (71.43)	4 (40.00)	2 (11.11)	3 (17.65)	
	4-7	1 (4.76)	6 (42.86)	11 (45.83)	13 (59.09)		1 (4.76)	3 (30.00)	10 (55.56)	8 (47.06)	
	>7	0 (0.00)	1 (7.14)	10 (41.67)	6 (27.27)		0 (0.00)	3 (30.00)	5 (27.78)	6 (35.29)	
History of positive PPD/Quantiferon		2 (9.52)	2 (14.29)	0 (0.00)	4 (18.18)	0.150[2]	2 (9.52)	2 (20.00)	0 (0.00)	2 (11.76)	0.318[2]
Diabetes		0 (0.00)	1 (7.14)	11 (45.83)	7 (31.82)	0.000[2]	0 (0.00)	1 (10.00)	7 (38.89)	5 (29.41)	0.005[2]
Metabolic syndrome		0 (0.00)	1 (7.14)	11 (45.83)	4 (18.18)	0.000[2]	0 (0.00)	1 (10.00)	7 (38.89)	3 (17.65)	0.006[2]
Cardiovascular Disease		1 (4.76)	1 (7.14)	11 (45.83)	9 (40.91)	0.001[2]	1 (4.76)	0 (0.00)	8 (44.44)	10 (58.82)	0.000[2]
Pulmonary Disease		5 (23.81)	4 (28.57)	7 (29.17)	7 (31.82)	0.972[2]	5 (23.81)	2 (20.00)	4 (22.22)	5 (29.41)	0.937[2]
CD4 count > 200			10 (100.00)		16 (94.12)	0.434[1]		10 (100.00)		16 (94.12)	0.434[1]
On ART			8 (80.00)		17 (100.00)	0.055[1]		8 (80.00)		17 (100.00)	0.055[1]
HIV VL > 100			8 (80.00)		17 (100.00)	0.055[1]		8 (80.00)		17 (100.00)	0.055[1]
% life span with HIV			33.5 (9.7-100)		26.1 (23..4-38.1)	0.850[3]		17.8 (8.3-38.5)		38.1 (22.2-50.0)	0.850[3]

[1]Chi Square.
[2]Fisher's Exact.
[3]Wilcoxon Rank Sum.

Metabolic syndrome = DM + HTN + HLD, PPD = purified protein derivative. All values are n (%). P values less than or equal to 0.05 were considered significant. The age groups include younger = 21-35, and (≥ 60 years) for most experiments (Monocytes). However, the age groups were extended to 21-40 and (≥ 50 years) for the Dendritic cell experiments.

Significance assessed using Chi Square[1] Fisher’s Exact Test[2] and Wilcoxon Rank Sum[3].

The value of % life span with HIV (since diagnosis) shown in the table represents an interquartile range, with the median (25 percentile, 75 percentile).

Supplementary Table 4. Multivariable regression model of IL-12 and TNF-α expression in CD11b+CD14+ activated monocytes.

A

Multiple linear regression results with least square means for IL-12 cytokine outcome in activated monocytes (N=81)

	Unadjusted model			Adjusted model- all variables		
	**Parameter estimate**	**Standard error**	**P-value**	**Parameter estimate**	**Standard error**	**P-value**
Age (Older vs Young)	19.90	3.79	<0.001	14.25	4.87	0.003
HIV (Positive vs Negative)	23.03	4.37	<0.001	13.99	6.19	0.024
Age x HIV interaction	-15.50	5.75	0.007	-9.68	6.05	0.110
Recreational Drug Use				1.06	4.40	0.810
History of Fungal Disease				4.10	6.78	0.545
Comorbid conditions				3.42	2.18	0.117
% lifespan with HIV				0.13	0.08	0.124

Least square means with 95% confidence intervals for interaction results

**Age/HIV status**	**Unadjusted model**			**Adjusted model- all variables**		
	**Mean**	**LCL**	**UCL**	**Mean**	**LCL**	**UCL**
Younger HIV negative	-8.61	-15.71	-1.51	-14.12	-19.54	-8.71
Younger HIV positive	5.38	-3.14	13.90	8.90	2.26	15.54
Older HIV negative	5.64	-0.75	12.03	5.77	0.70	10.84
Older HIV positive	9.95	3.72	16.18	13.30	8.00	18.59

B

Multiple linear regression results with least square means for TNF-α cytokine outcome in activated monocytes (N=81)

	Unadjusted model			Adjusted model- all variables		
	**Parameter estimate**	**Standard error**	**P-value**	**Parameter estimate**	**Standard error**	**P-value**
Age (Older vs Young)	8.75	3.30	0.008	8.78	4.15	0.035
HIV (Positive vs Negative)	14.20	3.81	<0.001	8.99	5.28	0.089
Age x HIV interaction	-4.05	5.01	0.419	-1.55	5.17	0.765
Recreational Drug Use				1.82	3.75	0.627
History of Fungal Disease				1.30	5.78	0.823
Comorbid conditions				-0.90	1.86	0.629
% lifespan with HIV				0.12	0.07	0.104

Least square means with 95% confidence intervals for interaction results

**Age/HIV status**	**Unadjusted model**			**Adjusted model- all variables**		
	**Mean**	**LCL**	**UCL**	**Mean**	**LCL**	**UCL**
Younger HIV negative	-14.49	-19.21	-9.76	-13.18	-19.24	-7.12
Younger HIV positive	-0.28	-6.07	5.50	-4.19	-11.45	3.08
Older HIV negative	-5.74	-10.16	-1.32	-4.40	-9.85	1.06
Older HIV positive	4.41	-0.20	9.03	3.05	-2.26	8.36

(**A**) Table of multivariable regression model for IL-12 expression in Activated Monocytes. (**B**) Table of multivariable regression model for TNF-α in Activated Monocytes. Tables outline the multivariable regression model with the following variables (Age, HIV status, Age/HIV interaction term), and the following covariates (history of recreational drug use, history of fungal infection, number of co-morbid conditions, and % HIV life span). LCL and UCL = Lower and Upper Confidence limit respectively.
